# Neutron Radiography Study of Laboratory Ageing and Treatment Applications with Stone Consolidants

**DOI:** 10.3390/nano9040635

**Published:** 2019-04-19

**Authors:** Matea Ban, Tim De Kock, Frédéric Ott, Germana Barone, Andreas Rohatsch, Simona Raneri

**Affiliations:** 1 Institute of Geotechnics, Research Centre of Engineering Geology, Vienna University of Technology, 1040 Vienna, Austria; andreas.rohatsch@tuwien.ac.at; 2 Department of Geology, Ghent University, 9000 Ghent, Belgium; Tim.DeKock@UGent.be; 3 Laboratoire Léon Brillouin, Université Paris-Saclay, Centre d’Etudes de Saclay, 91191 Gif sur Yvette CEDEX, France; Frederic.Ott@cea.fr; 4 Department of Biological, Geological and Environmental Sciences, University of Catania, 95129 Catania, Italy; gbarone@unict.it; 5 Department of Earth Sciences, University of Pisa, 56126 Pisa, Italy; simona.raneri@unipi.it

**Keywords:** neutron radiography, stone consolidation, treatment application, artificial ageing, water absorption, brushing, poultice, capillary absorption

## Abstract

A nano-silica consolidant and nano-titania modified tetraethyl-orthosilicate were applied on two building stones, a carbonate and a silicate, by brush, poultice or capillary absorption. Neutron radiography was used to monitor capillary water absorption, and to analyse changes in physical properties caused by heat treatment of specimens for the purposes of artificially ageing and different treatment applications with stone consolidants. Moreover, ultrasonic pulse velocity and gravimetrically determined water absorption were analysed to cross-validate neutron radiography. The results reveal that reactive systems like tetraethyl-orthosilicates need an unknown period for polymerisation, which makes nano-silica consolidants more favourable for construction follow-up work. While polymerisation is incomplete, hydrophobic behaviour, water trapping and pore clogging are evident. Within the tetraethyl-orthosilicate treatment, poultice and brushing are strongly influenced by the applicant, which results in wide ranging amounts of water absorbed and anomalous water distributions and kinetics. The carbonate lithotype displays polymerisation initiated in the core of the specimen, while the lateral surfaces are still mostly hydrophobic. Reaction time differences can be attributed to the different amounts of consolidants applied, which is a result of the chosen application settings. Artificial ageing of stone specimens is a prerequisite when mechanical strength gain is studied, as demonstrated by sound speed propagation.

## 1. Introduction

In the field of built cultural heritage, the preservation of monumental stone represents a challenging task. Basically, preservation can be divided into the protection and the consolidation of stone materials, which are accomplished by applying materials onto the stone surface. In the case of protection, products are applied to prevent harm from environmental factors, while in the case of consolidation, they are used to restore the mechanical strength of the stone by re-establishing the lost grain cohesion. As natural stones vary, so do their decay patterns. The vast majority of these decay phenomena are summarised in the ICOMOS-ISCS glossary on stone deterioration patterns [1]. A great number of these phenomena exhibit a necessity for consolidation. The most important of such phenomena is disintegration, which can further be subdivided into crumbling and granular disintegration, where the last includes decay patterns like powdering or chalking, sanding and sugaring. Moreover, deterioration patterns like peeling and flaking might also require consolidation. In certain cases, micro fissures induced through differential stresses may be consolidated in order to compensate for the lost mechanical strength. However, all those phenomena occur to a different extent, complexity and can be accompanied by material loss (e.g., loss of the binding matrix, rounding, roughening, etc.). The cause and development of such phenomena is beyond the scope of the present study, and, in most cases, the decay patterns that are in need of consolidation are additionally overlapped with other decay phenomena, e.g., biological colonisation, cracks or deformation, that are in need of additional restoration actions. For such a diversity in substrates and conditions, new products with multi-functional self-cleaning [2], anti-fouling [3], water repelling [4] or anti-fungal [5,6] properties are continually being developed, with the aim of incorporating functionalised systems in consolidants. With such a range of consolidating materials in terms of chemical, physical and mechanical characteristics, the efficiency and compatibility of these treatments are likely to vary. Therefore, study of the physico-mechanical interactions between the substrate and different consolidating materials seems mandatory, in order to prevent eventual harmful effects caused by the treatment itself. It is known that the efficiency and compatibility of a treatment strongly depends on the interplay of several factors [7,8], including the substrate features and its conservation state, application methods and, as already mentioned above, the consolidating product itself.

Porosity and pore geometry of natural stones modified by weathering processes result in different pathologies. To better understand these decay patterns, artificial ageing of stone materials is frequently employed prior to applying and testing stone consolidants. The emphasis lies in the development of micro-cracks caused by differential stresses, which are induced through heat treatment [9,10]. However, several other laboratory test methods exist that supposedly mimic natural weathering decay phenomena, like salt crystallisation, thermal stresses, freeze–thaw and chemical weathering, to name a few. Specific protocols of accelerated aging are difficult to recommend because of the manifold dependency of different lithotypes, with varying degrees of resistance towards the induced stresses, leading to difficulties in adjusting the ageing protocol [11].

Regarding the protocol of treatment in monumental stone consolidation, three application methods—namely brushing, poultice and partial immersion—are commonly employed [12,13]. Brushing seems to be the preferred application technique in Italy, as reported in the literature [14]. It allows control of the amount of consolidant applied, and thus possibly assures reproducibility of the treatment procedure. However, treatment application varies and every country favours its own technique, mostly based on traditions and common local practice. Other application techniques not often discussed in the literature, but still widely employed in stone consolidation, include methods like run-off with different utensils (such as wash bottles, syringes and pipettes), spraying or pumping with different pressures, capillary absorption, vacuum pumping and injections through boreholes [15,16,17]. It must be pointed out that the differences in application routines also relate to different conservation problems that need to be managed (i.e., surface treatment or structural consolidation associated with larger penetration depths) [18]. For example, if a stone exhibits decay patterns in the form of sugaring or sanding, brushing will not be the method of choice, as it would remove an unknown amount of original material before consolidating it. On the other hand, if a dense surface is followed by a disintegrated subsurface zone, drilling boreholes to reach the material that needs to be consolidated is a prerequisite to remedying the problem. General rules or guidelines for treatment applications are difficult to assess, because of the manifold dependency of different stone types and their decay patterns.

As natural stone can accommodate different amounts of consolidants, and consolidants are used in different concentrations of solid content, it is necessary to tailor those variables in order to study the possible advantages and disadvantages connected to treatment performance. In addition, treatments implemented with different application techniques will most probably result in different amounts of product being applied, and thus different treatment performances. It has already been confirmed that treatment performance depends on the amount of product applied [19] or the number of application cycles used [20]. In laboratory studies, treatment application is usually time-controlled, by allowing a building material to absorb the consolidant within a defined time span. On-site, it is common practice to apply the consolidant until refusal or full saturation of the substrate is observable. The latter application practice will result in a higher amount of product absorbed than when a consolidant is applied by, for example, a few brush strokes. The penetration depth might also differ when using different application techniques, as in immersion of all surfaces versus absorption from one surface.

The application of nanotechnology has not bypassed the field of architectural preservation. In fact, nano-particle based products have been emerging in recent years with the aim of tackling problems that have not been solved yet in stone conservation. Due to their nano-metric size, the consolidating materials are said to be more reactive and possibly better able to adhere to the surfaces, able to penetrate deeper and into smaller passages, and chemically more compatible with the host substrate, as well as protecting the building material from atmospheric pollution and further environmental agents. The main inorganic nanomaterials include calcium hydroxide and calcium carbonate systems [21,22,23], different metal oxides [24,25] and colloidal nanosilica [26], either used solely or incorporated into a matrix to obtain a particle-modified consolidant [27]. As the materials are new, many aspects of their use and durability remain unknown. It is certain that these systems will engage many further conservation scientists to come, especially as the use of such systems is not only limited to mineral materials but also regards materials such as wood [28] or paper [29].

Across the large range of chemically different stone consolidants, tetraethyl-orthosilicates—better known as TEOS—are the most widely used group. Through a step-wise polymerisation, a silica gel is formed by hydrolysis and condensation that, in consequence, consolidates the substrate. TEOS have been modified for different purposes, including with regards to the application procedure. Dilution of the consolidant was a common practice to reduce over-consolidation [30]. For the application method known as the vacuum-circling process [31], used to enhance penetration depths, specially tailored products have been engineered by Remmers (Löningen, Germany) [32]. The main purpose of the modification was to facilitate the process of hydrolysis. Enhancement of hydrolysis was desired because the consolidant could remain liquid in the stone material for an unknown period.

Any consolidation will ultimately change the stones’ petrophysical properties (e.g., reduction of capillary water absorption or changes in water vapour permeability, shift in the pore radii distribution, changes in modulus of elasticity, etc.). For building materials in general, modifications of water transport and retention are particularly relevant. Moisture represents the most common cause of damage in monumental stones, therefore, change in water absorption by capillarity is an important property to be studied. To evaluate changes in water absorption before and after a treatment, laboratory and on-site tests are often employed [33,34]. However, a non-invasive method, such as neutron imaging, seems to be an adequate technique to fully investigate aspects related to local distributions of water quantities and kinetics. This method goes beyond simple visual inspections of advancement fronts of water, and the gravimetrical quantification of water absorbed [35]. Furthermore, neutron imaging allows assessment of the penetration depth and distribution of products inside the material [36,37], and it provides a direct non-destructive evaluation technique where the same specimens can be used for further analysis.

Different natural stones, their varying decay patterns, numerous consolidating materials and application techniques employed result in many variables that are responsible for the outcome of a treatment performance. Research is lacking on all these variables, and they are thus not fully understood. In view of this, the goal of the present experimental study is to analyse how water absorption by capillarity is influenced when different consolidants are applied by different techniques. Through this, an understanding of the possible advantages and drawbacks of certain application techniques and consolidants can be gained, which is crucial for on-site work on monuments. For this purpose, neutron radiography has been used at the *IMAGINE* beamline, located at the Laboratoire Léon Brillouin (LLB), at the Orphée Reactor in Saclay, France [38]. This imaging technique allowed monitoring of the water absorption by capillarity in two different porous stones, consolidated with two different products applied by three application techniques. The two newly developed consolidants are a nano-silica suspension in a mixture of water-ethanol, called NC-12C, and a nano-titania particle modified tetraethyl-orthosilicate in isopropanol called NC-25C. Both products were developed as part of the European project named “*Nano-Cathedral*” (Grant Agreement No. 646178) [39]. The consolidants were applied by brush, poultice or capillary absorption. Depending on the mode of application, different amounts of product were applied, allowing us to assess whether possible changes of material properties might be related to the treatment application itself, as well as the effective amount of solid content after curing that is precipitated in the stone material. Furthermore, a specific task of the present study included the analysis of the early stage of consolidant application, as this represents a crucial time step and determines the follow up work on the construction site.

The results demonstrate that the application technique, and therefore the amount of product absorbed, are relevant when reactive systems like TEOS are used. The amount of consolidant applied also governs the speed of polymerisation. The apparent hydrophobicity, present due to incomplete polycondensation even six weeks after application, causes pore clogging and water trapping. Poultice and brushing methods are strongly influenced by the applicant, which results in wide ranging levels of water absorption and anomalous water distribution and absorption kinetics within specimens treated with TEOS. This study reveals that, in the case of the carbonate lithotype, while the lateral surfaces remain mostly hydrophobic, polymerisation initiated in the core of the specimens might be present. Nano-silica-consolidated stone displays homogenous water absorption regardless of the amount of product applied and application technique used, which presents a clear advantage of such systems for on-site use. For studies concerning the mechanical strength gain, artificial ageing of stone specimens should be a prerequisite. While the cross-validation of neutron imaging with ultrasonic pulse velocity and water absorption coefficient was successful, insights into water trapping and local anomalies were only possible through neutron radiography.

## 2. Materials and Methods

### 2.1. Characterisation of the Lithotypes

**Figure 1:**
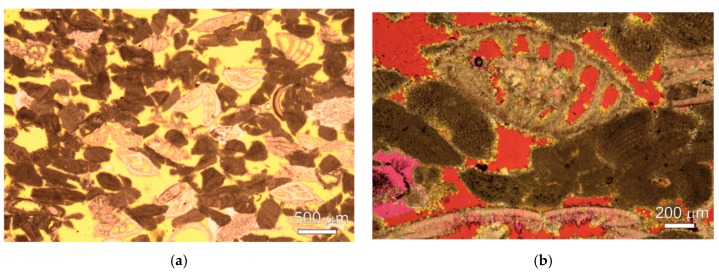

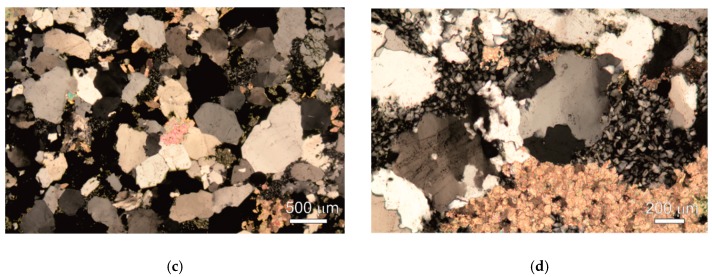
Micrographs of the stone fabric taken with a polarized light microscope: (**a**) Fabric of St. Margarethen Limestone with nearly equally distributed debris of coralline red algae and rotaliide foraminifers; (**b**) detail of the so-called dogtooth cement displayed as yellow binder between the microfossils; (**c**) fabric of Schlaitdorf Sandstone mainly consisting of quartz beneath feldspar and carbonate (pinkish colour), with planar to lobate grain boundaries, and (**d**) detail of the grain boundaries, the kaolinite matrix and sparitic carbonate.

Two porous sedimentary stones, a carbonate and silicate, were characterised by polarised light microscopy (PLM, Zeiss, Italy). The selected stones exhibited different mineralogical compositions, textures and structures, which were important prerequisites for this study in order to distinguish the possible influences a stone material can have on treatment applications. The petrographic differences of both lithotypes can be observed in Figure 1a–d. Results and a discussion concerning the mercury intrusion porosimetry of both lithotypes can be found in a previous study [40].

Miocene (Langhian) calcareous arenite, known as St. Margarethen Limestone, can be classified as a porous grainstone or biosparite [41]. This limestone has been extracted from Roman times through to today in the quarry of St. Margarethen in Burgenland, Austria. It mainly consists of debris from coralline red algae, beneath foraminifers, and debris of echinoids and bryozoans, as well as traces of quartz and muscovite from the metamorphic geological hinterland. The cementation can be classified as early diagenetic fine-grained dogtooth calcite. The grainsize distribution can be described as nearly equigranular and moderate to well sorted. The mean grainsizes range from 0.5 to 1 mm, and the total porosity is between 25 to 30 vol.%. Additionally, larger fossils such as rhodolithes, oyster shells and skeletons of sea urchins up to several centimetres in size can be observed.

The Triassic (Keuper) lithotype, known as Schlaitdorf Sandstone, can be classified as a porous medium-grained siliciclastic arenite, which has been quarried since Roman times through to today in the province of Baden-Württemberg in Germany. This sandstone mainly consists of quartz (~72%), feldspar (~2%) beneath dolomite spar and calcite (~8%) and lithic fragments (~12%), as well as a clayey matrix which mainly consists of kaolinite (~6%) and traces of illite. The grainsize distribution can be described as well sorted, with an average grainsize of about 0.5 mm. The mean pore radius is about 3 µm, with a total effective porosity of approximately 20 vol. % [42].

Both lithotypes have been extensively used in construction of emblematic buildings across Austria and Germany, respectively. While Schlaitdorf Sandstone is still used for construction purposes, St. Margarethen Limestone is, in the present day, primarily used for restoration work. Their decay patterns range from surface deteriorations to structural disintegration, and both varieties are commonly in need of consolidation, since they are prone to weathering.

### 2.2. Stone Consolidants

The stone consolidants selected for the present study are newly designed products, developed in the frame of the European Horizon 2020 funded *Nano-Cathedral* project, short for “Nanomaterials for conservation of European architectural heritage developed by research on characteristic lithotypes” (Grant Agreement No. 646178). The project’s goal was to develop new, and modify existing, materials for the protection and consolidation of monumental stone. As many developed products never make it to the market, the project call for nanomaterials, advanced materials, and production (NMP-21-2014: materials-based solution for protection or preservation of European cultural heritage) aimed at large-scale production and application of the newly developed materials. Innovative SMEs (small and medium-sized enterprises) and industries have been partnered to achieve this, as the designed products require a high technological readiness to enter the market after successful completion of the project, which is why the synthesis routes of the materials are protected by a non-disclosure agreement. Two newly developed products were tested in the present study.

The first product, NC-12C, is a silicon dioxide nanoparticle consolidant in suspension in a neutral water–ethanol mixture. The consolidant appears as a milky liquid with a low viscosity. The active ingredient is the suspended silica nanoparticles that make up 17 wt. %, and their average dimensions are 35 nm. The density amounts to 1 kg/L and the viscosity to 2 mPa s (both determined at 25 °C). If the consolidant is stored in a cool, dry place, the shelf life is at least one year. The product was developed by the Italian based industry Colorobbia S.p.A. (Sovigliana-Vinci (Firenze) Italy). Nanoparticle-based products are emerging more and more, as evidenced by recent publishing activities in the field of built cultural heritage [43]. These materials can be beneficially tailored in terms of dimensions, concentration, and use of solvent, to name just a few properties, all of which determine treatment performance. As soon as the solvent evaporates, the deposited nano-particles are structured into a solid material that is supposed to strengthen the stone.

The second product, NC-25C, is a tetraethyl-orthosilicate in isopropanol with 70% active content and TiO_2_ dispersed particles. The spherical TiO_2_ particles come in two crystal phases, namely anatase (approximately 80%) and rutile (approximately 20%). Approximately 1% added titania has a positive effect on the self-cleaning abilities of the consolidant, which can be considered an additional value when compared to commercial TEOS. The average size of the nano-titania is 10 to 15 nm. The consolidant appears white and has a low viscosity. The specific weight is ~1 g/cm^3^, while the viscosity is 3,3 cSt (both determined at 25 °C). The reaction takes place after four weeks at room temperature (~20 °C) and with a relative humidity of 45 ± 5%. If the consolidant is stored in a cool, dry place, the stability of the product is at least six months. The consolidant was developed by the Italian industry Chem Spec S.r.l. while the supplier of the nanoparticle is the Spanish based industry Tecnan (Technologia Navarra de Nanoproductos, S.L., Los Arcos (Navarra) Spain). TEOS are favoured for their low viscosity and thus good penetration depths, chemical resistivity and induced mechanical strength. As nano-particle based consolidants, TEOS can also be tailored to obtain different properties. This is achieved through the pre-polymerisation degree, addition of solvents, admixtures of catalysts, ratio of different mono- to oligomers, or the addition of compounds to gain more elasticity, better adhesion to carbonate substrates, and so forth. According to the manufacturers of the products, the ideal condition for polymerisation is four weeks at a temperature of 20 °C and a relative humidity of 50%. During the curing period, the system is known to possess hydro-repellent properties. The silica gel formed in the pores of the substrate undergoes continuous drying accompanied by shrinkage over an unknown time span.

### 2.3. Artificial Ageing of Stone

Cyclic heat treatment was chosen as an artificial ageing technique, as it showed sufficient ability to cause a reduction of soundness. The aim of the ageing was to cause microstructural defects in the form of micro cracks, and to observe the alterations caused by the cracks to the stone’s capillary water absorption. Two main reasons motivated the use of temperatures as high as 600 °C. The first reason is the porosity of the stone, where higher temperatures needed to be employed to assure sufficient microstructural damage. In the case of St. Margarethen Limestone, large pores and microfossils are able to accommodate more stresses during thermal expansion, which is why higher temperatures and cyclic stresses were preferred. The second reason is that, for the silicate variety Schlaitdorf, only temperatures above 573 °C cause sudden volumetric expansion when transforming from α- to β quartz, which results in thermal expansion and subsequent micro cracking.

The stones were heat treated in an electric furnace of 3.5 L volume from Thermo Scientific (Fisher Scientific Austria GmbH Vienna), model Heraeus K 114. The heating rate was 40 °C/min until a static temperature of 600 °C was reached and maintained for one hour. This procedure was repeated three times, with a cooling of the specimens between the cycles. To guarantee the same starting point, all samples were washed and dried to a constant weight before the consolidation treatment.

### 2.4. Consolidation of Stone: Treatment Procedure and Curing

**Figure 2:**
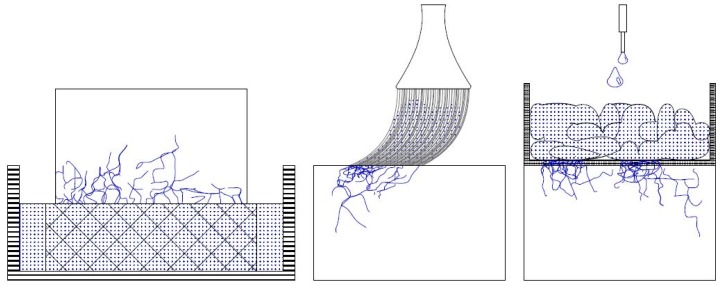
Schematic representation of application techniques used to consolidate stone specimens. Capillary absorption for one hour (**left**), brushing with 10 strokes (**middle**), and poultice, where a cellulose pulp packed in Japanese paper was placed on the stone surface for 1 h (**right**).

In total, 60 specimens with dimensions of 2 cm × 2 cm × 5 cm were washed with deionized water, dried at 50 °C until they reached a constant mass and kept under laboratory conditions for 24 h before the treatment application. The dry mass of the stone specimens was recorded before the treatment procedure. For each studied condition (sound, aged and consolidated), three specimens were used for both lithotypes (see Table 1). The consolidants were applied by capillary absorption, brushing and poultice (see Figure 2).

Treatment by capillary absorption was performed by placing the samples in contact with the consolidants for one hour. The specimens were placed on a wide meshed plastic grid, allowing the absorption of the products to take place from one side by capillary forces. The consolidant was refilled to assure a constant contact with the treated surface, making sure that no immersion of the specimen occurred through the lateral sides of the specimens. For such purposes, a wider box filled with consolidants was suitable. In the case of capillary absorption treatment with the product NC-12C, pre-wetting with ethanol was conducted. The aim of such pre-treatments was inspired by on-site work. Apparently, a pre-treatment using the corresponding solvent of the product allows a deeper penetration of the consolidants into the stone and avoids glossiness through particle efflorescence at the surface. Moreover, a so-called over-consolidation in the case of the capillary absorption specimens treated with the product NC-25C was mimicked by repeating the procedure three days in a row. In the case of over-consolidation, the sample was left under laboratory conditions between the application cycles (T: 20 °C ± 3 °C and RH: 55% ± 3%), where a chemical reaction might already have started. The aim of such a procedure was to study the impact of over-consolidation [20]. According to technical guidelines, such an application procedure should be avoided, as pore clogging may occur due to the already-started polymerisation. The right approach when working with reactive consolidants like TEOS is to do the treatment wet-in-wet to avoid such phenomena as pore clogging and accumulation of the consolidant into the sub-surface zone. In the present work, the goal was to study, indirectly, through the absorption of water, if such an over-consolidation would retard the polymerisation.

Treatment application by brushing was done following the recommendations reported in the literature [44], by a continuous application of 10 strokes onto the stone surface. Concerning consolidation with NC-12C, one set of specimens was pre-wetted with ethanol, while a second set was consolidated without pre-wetting. The aim of such a procedure was to compare the possible influences of the pre-treatment on the behaviour of water absorption by capillarity. Pre-wetting with ethanol was completed by brushing the surface 10 times in a row.

For treatment application by poultice, it was applied following procedures reported elsewhere [12]; a cellulose pulp soaked with the consolidating products was stirred for two hours prior to application onto the stone surface. During stirring, evaporation of the solvent was avoided by the use of a sealed container. A weight ratio between cellulose pulp and consolidants of 1 to 8 was mixed. The layer of the consolidant-soaked cellulose pulp which came in contact with the stone surface was approximately 2 cm thick. The poultice was wrapped in a so-called Japanese paper that was subsequently placed in contact with the stone surface for one hour. The Japanese paper was used to avoid direct contact of the stone with the cellulose derivate, to avoid gluing or even soaking of the pulp into the substrate. For the application of NC-12C, specimens were pre-wetted with ethanol by stirring the solvent with the cellulose pulp for two hours, and subsequently placing the mixture for one hour on the surface of the sample.

After treatment, samples were weighed, placed in a sealed box and wrapped for 48 h in a plastic foil to prevent quick evaporation and back-migration of the consolidants. The next step was outdoor curing, which took place in a sheltered area in the courtyard of the Department of Earth Sciences at the University of Pisa in Italy. The consolidated specimens were gravimetrically monitored every day for three weeks, and the environmental conditions were recorded once per day in the early afternoon (see Figure S1 in Supplementary Materials). The daily temperatures ranged from 17 °C to 28 °C, while the relative humidity varied between 30% and 85%. It must be noted that a day to night fluctuation of the temperature and relative humidity during the curing period was not recorded. The values recorded can be viewed as quasi-ideal for curing conditions (according to common technical sheets, 20 °C and 50% RH for three to four weeks), but it must be emphasised that the temperatures dropped during the night.

Prior to neutron radiography studies, the stone specimens were stored under laboratory conditions for three additional weeks, which means that the first scans were determined six weeks after treatment applications.

### 2.5. Neutron Radiography and Image Processing

Neutron radiographs were acquired at the instrument *IMAGINE* of the Laboratoire Léon Brillouin in Saclay, France. A neutron beam was sent onto the samples. Some of the neutrons were absorbed or scattered. One measures the intensity of the so-called transmitted beam—the part that crosses the sample without interacting with it. The flux of the incident beam was 2 × 10^7^ neutrons cm^−2^ s^−1^. It was a so-called white beam that included a large spectrum of cold neutrons, with a wavelength in the range of 3 to 20 Å. The optical setup was defined by the L/D ratio of 400. The L/D ratio (ratio of L being the distance between the entrance aperture of the beam to the image plane over D that is the diameter of the beam aperture) represents a key factor determining the quality of the resolution [45]. The bigger the L/D ratio, the lower the angular resolution of the neutron beam and the better the resolution. Detection was achieved using a sCMOS camera (Andor) coupled with a lithium scintillator of 100 µm thickness. This set-up allowed a spatial resolution of about 250 µm. In our case, the main contrast was due to hydrogen atoms being more present in water than in the samples. The cross section of a hydrogen nucleus is almost two orders of magnitude larger than those of the majority of nuclei, including those of the stones. For details regarding a closer description of the technique and the use of neutrons in cultural heritage, the reader is referred to References [46,47] for further information.

Dark field and open beam images were first acquired and used for further corrections. Stone specimens were placed on a stack of filter paper and arranged in an aluminium container so that the whole stone specimens, from top to bottom, could be scanned. Reference images in dry state were acquired. To monitor the water absorption by capillarity, water was manually added into the aluminium box, which ensured the saturation of the filter paper pack (with a 10 mm thickness, obtained from Kaltek S.r.l. Padova Italy). After the addition of the water, scans were acquired with an exposure time of 10 s until full saturation, or after a maximum duration of 30 min.

The images were pre-processed according to the in-house protocol at the *IMAGINE* beamline, performing a correction with dark-field images, normalization with open beam images and noise filtering (dead camera pixels and spurious gamma events). Absolute transmission images of the samples were obtained with an absolute precision in the 1% range. Neutrons, being strongly scattered by hydrogen atoms, with equivalent thicknesses of 20 µm of H_2_O in a sample, can be detected in favourable cases. To analyse the absolute water content, water absorption images were normalised with the steady-state dry images taken prior to absorption, to obtain images in which the measured transmission was directly related to the amount of water in the sample [48]. A similar approach was performed on a staircase-like sample holder containing twelve different water volumes with standardised thickness. The neutron transmission was plotted over these twelve water thicknesses from 0.09 mm to 5.00 mm and used as a calibration curve for the water content of the water absorption images. This procedure was necessary because for high water contents (>2 mm equivalent thickness), the transmission does not follow a simple Beer–Lambert law. Pre-processing and image analysis were performed using Fiji ImageJ (Fiji Is Just ImageJ - Image Processing and Analysis in Java, open source, general public license) [49].

### 2.6. Ultrasonic Pulse Velocity and Water Absorption Coefficient

Two additional non-destructive natural stone test methods were carried out in the laboratory to investigate a possible cross-validation for the use of neutron radiography. For this purpose, the determination of the ultrasonic pulse velocity (UPV) and water absorption coefficient (WAC) were used on the same specimens that were studied by means of neutron radiography. Three specimens for each condition were analysed.

The velocity of propagation of pulses of ultrasonic waves was determined according to the standard EN 14579 [50]. The device used consisted of an electrical pulse generator (type Conosonic C2-GS) and a pair of transducers (UP-DW, with a diameter of 24 mm but a coupling surface as a probe tip), all developed by Geotron-Elektronik (Pirna, Germany).UPV is reported in [km/s], and the frequency used for both stones amounted to 80 kHz, while the amplitude was selected according to samples damping; for the studied stones, it was between 200–500 mV.

The water absorption coefficient was determined after 30 min (WAC_30_). The specimens were in direct contact with the wet filter paper (obtained from Kaltek S.r.l. Padova Italy ), and the weight increase was recorded at time intervals of 1, 3, 5, 10, 15, 20, 25 and 30 min. The surface that was in contact with water was the surface that was treated by the consolidants. It is important to note that, for porous stones, the slope of the curve represents the speed of adsorption and the plateau of the curve represents the full saturation of specimens, and these two phenomena are not distinguished in WAC_30_. In order to differentiate between these two processes, WAC_5_ was also introduced, which represents the water absorption coefficient after 5 min. The latter coefficient solely considers the speed of the water absorption, or the initial part of the curve. Both coefficients, WAC_30_ and WAC_5_, are reported as [kg/(m^2^·*t*^0.5^)].

UPV and WAC were tested eight weeks after the treatment applications. It must be stressed that, in the latter case, the consolidated specimens already came in contact with water during neutron radiography tests and that, therefore, the polymerisation was at an advanced stage.

## 3. Results and Discussion

### 3.1. Evaluation of Artificial Ageing

**Figure 3:**
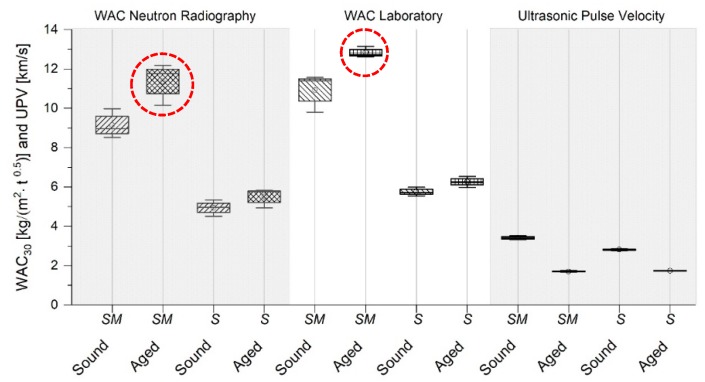
The effect of thermal ageing on St. Margarethen Limestone (**SM**) and Schlaitdorf Sandstone (**S**) determined by the water absorption coefficient after 30 min, as well as the ultrasonic pulse velocity before (**sound**) and after artificial ageing (**aged**). WAC was calculated from image analysis obtained from neutron radiographs and gravimetrical laboratory analysis. The corresponding values can be extracted from Supplementary Materials, Table S1.

**Figure 4:**
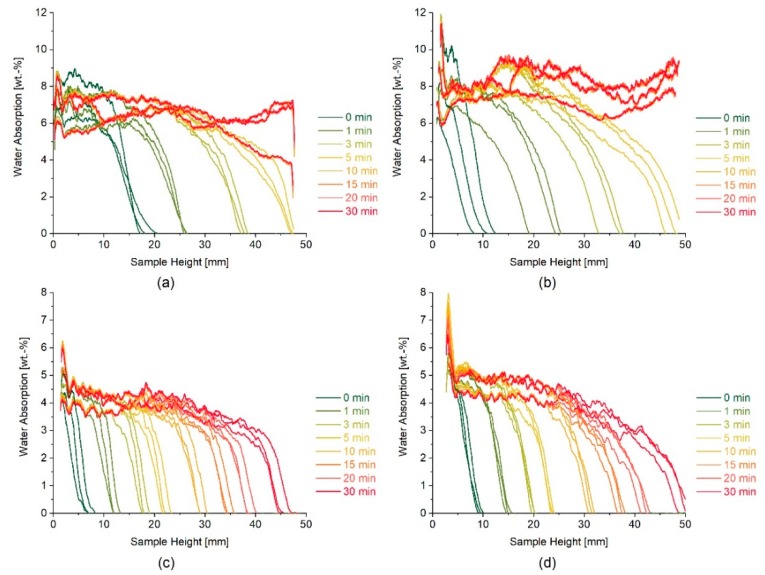
Neutron studies of sound (**a**) and artificially aged (**b**) water absorption kinetics (reported as wt. % and as a function of the sample height at intervals of 1, 3, 5, 10, 15, 20, 25 and 30 min) for three stone specimens of St. Margarethen Limestone, as well as sound (**c**) and artificially aged (**d**) water absorption curves of three stone specimens of Schlaitdorf Sandstone.

The impact of heat treatment on artificially aged stone substrates prior to consolidation was successfully evaluated by neutron radiography, the water absorption coefficient and the ultrasonic pulse velocity (see Figure 3 and Figure 4).

In the case of highly porous materials, the WAC sometimes displays marginal differences between sound and artificially aged substrates, due to the limits of accommodating water. That is, with only a few induced micro cracks, the magnitude of absorbed water might be negligible in terms of weight increase. For such cases, the initial slope of water absorption curves can give more insight into fabric alterations and the speed of absorbed water than the water absorption coefficient itself. Franzoni et al. [9] have already demonstrated that, for some lithotypes, the initial part or the slope of the curve remains unchanged, because this part is associated with the size and amount of coarser pores, while the second part of the curve increases because the latter part is linked to smaller pores, especially those induced by heat treatment. Following these considerations, Table 2 gives the difference between water absorption coefficients calculated after 30 min and 5 min from both neutron radiographs and gravimetrical increases. In the case of the more porous lithotype, St. Margarethen Limestone, the difference between these two coefficients is apparent. As for Schlaitdorf Sandstone, the values are significantly close, because this stone does not reach the saturation equilibrium like St. Margarethen. St. Margarethen Limestone is a typical example of a one-dimensional Fickian diffusion process, where the initial part of the curve displays a linear increase of water absorption with the square root of time until it reaches a plateau, which represents the saturation of the specimen. Schlaitdorf’s curve also gradually increased with a linear dependency, but no equilibrium plateau can be reached for the given test time.

Figure 3 displays the trend of the WAC_30_ calculated through neutron radiography, and WAC_30_ obtained in the laboratory by gravimetrical means. The WAC_30_ values calculated through neutron radiographs are slightly underestimated in magnitude due to different effects, including: (i) averaging during image analysis, which also assumes a mean density and uniform specimen dimensions, (ii) crop of the bottom of the radiographs for reasons of shift corrections and (iii) effects considering the ratio of the signal to noise, timing and setting of the instrument, which might also include evaporation phenomena. Nevertheless, it was demonstrated that the artificially aged stone had an increase in water absorption by capillarity and a decrease in ultrasound pulse velocity, caused by the formation of micro cracks. Furthermore, in the case of St. Margarethen Limestone, a wider spread of the results is visible when the WAC_30_ was calculated from radiographs, indicating a more inhomogeneous distribution and speed of water absorbed in the artificially aged specimens (see Figure 3, data marked in red). UPV is suitable for the detection of micro cracks, especially when sound reference values are available.

Neutron radiography was able to detect changes in the speed and amount of water absorption in the case of both lithotypes due to the inducement of micro cracks. The speed of capillary suction was slightly higher for aged compared to sound stone specimens (Figure 4a–d). In the case of St. Margarethen Limestone, after ageing there was a wider spread in kinetics of absorbed water, indicating that the induced micro cracks resulted in a more inhomogeneous substrate. On the contrary, in the case of Schlaitdorf Sandstone, the recorded speed was more uniform after artificial ageing because of the induced cracks (compare Figure 4c,d).

Changes caused by heat treatment had a measurable impact on water absorption kinetics in both lithotypes, but for practical considerations and because the lithotypes are porous enough even in their sound states, the treatments were performed on sound stone specimens. However, neutron radiography proved to be useful when studying different pathologies of the stones’ fabrics caused by artificial ageing. Moreover, as will be demonstrated below, the impact of thermal treatment on the mechanical properties of the stone is a requirement for studies regarding mechanical strength gains, and thus treatment efficiency.

### 3.2. Evaluation of Treatment Application with TiO_2_ Modified Tetraethyl-Orthosilicate on St. Margarethen Limestone and Schlaitdorf Sandstone

**Figure 5:**
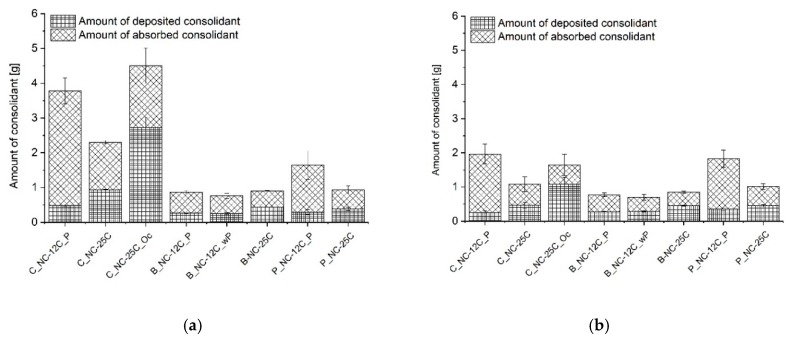
Comparison of the amount absorbed versus amount deposited inside (**a**) St. Margarethen Limestone and (**b**) Schlaitdorf Sandstone. For the calculation of the absorbed amount of consolidant, the weight of the stone in the initial dry state was subtracted from the weight after consolidation. To calculate the deposited amount of consolidant, the cured stone was weighed and subtracted by the weight of the initial dry state. The cured state corresponds to the mass recorded at the last day of outdoor curing, three weeks after the treatment application.

**Figure 6:**
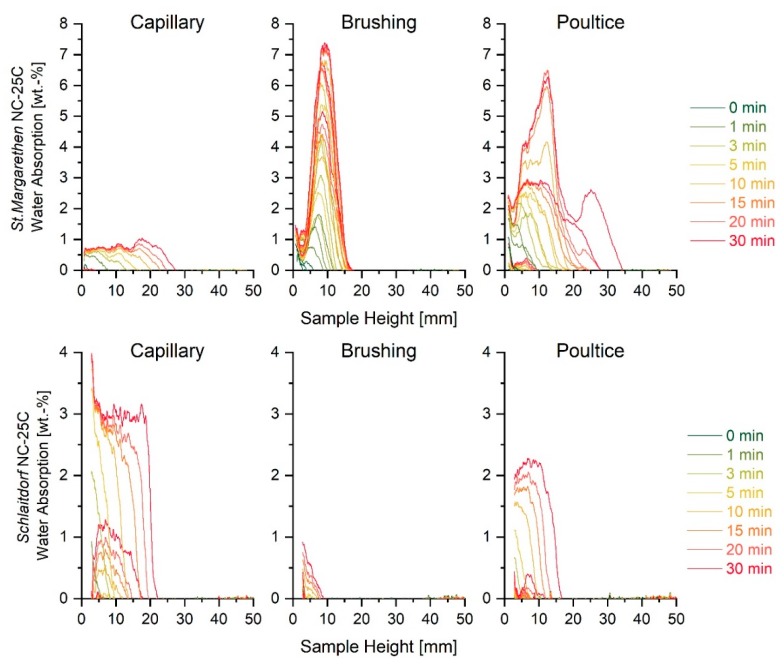
The top graph displays water absorption kinetics reported in weight percentage for three St. Margarethen Limestone specimens treated with NC-25C, while the bottom graph displays the same for Schlaitdorf Sandstone.

In the case of St. Margarethen Limestone, the acquired experimental data show that for the consolidant NC-25C, the reaction kinetics are influenced by the amount of product applied, and therefore it is application treatment dependent. Neutron radiographs clearly demonstrate that six weeks after the treatment application, the system was still largely hydrophobic. Derived from residual ethoxy groups, the system remained water repellent for an unknown period of time, depending on numerous factors. This apparent hydrophobicity disappears with continued hydrolysis–condensation reactions, and can even be overcome by a post-treatment with water, as demonstrated by Franzoni et al. [51]. When in contact with liquid water, the surfaces become less water repellent as hydrolysis proceeds. However, a long-lasting partial hydrophobicity might still be present after years, as shown in previous work [52]. It was determined that this effect of temporary hydrophobicity was most pronounced in the set of samples treated by capillary absorption, where the highest amount of product was deposited inside the stone’s fabric (see Figure 5a). Stone specimens treated with brushing and poultice accommodated a smaller amount of product due to the given application settings, thus allowing, on average, a faster polymerisation reaction. It is important to note that it is hardly possible to make assumptions about competing processes of hydrolysis and condensation in the period after initial treatment with neutron radiography, as it cannot provide direct evidence of polymerisation (consumption and production of water), but indicates indirectly the chemical process through which the water is absorbed. A couple of phenomena, not able to be distinguished with the present methodologies, might now be relevant in understanding the polymerisation process in a stone fabric in respect to water absorption: i) capillary flow in mixed patterns of hydrophobic–hydrophilic surfaces, where the latter might correspond to unconsolidated surfaces or already polymerised residues, ii) influence of the compositional and micro structural features of the stone fabric, including effects like surface area to volume ratio of the deposited consolidant [30], surface polarities, effects like vapour pressure, steric hindrance and inductive effects, and iii) dynamic changes occurring due to direct contact with liquid water during the tests. Even without further clarification of the above-mentioned phenomena, neutron radiographs acquired for sound and consequently consolidated stone allowed evaluation of what kind of impact a treatment application might have with respect to capillary water absorption (see Figure 6).

**Figure 7:**
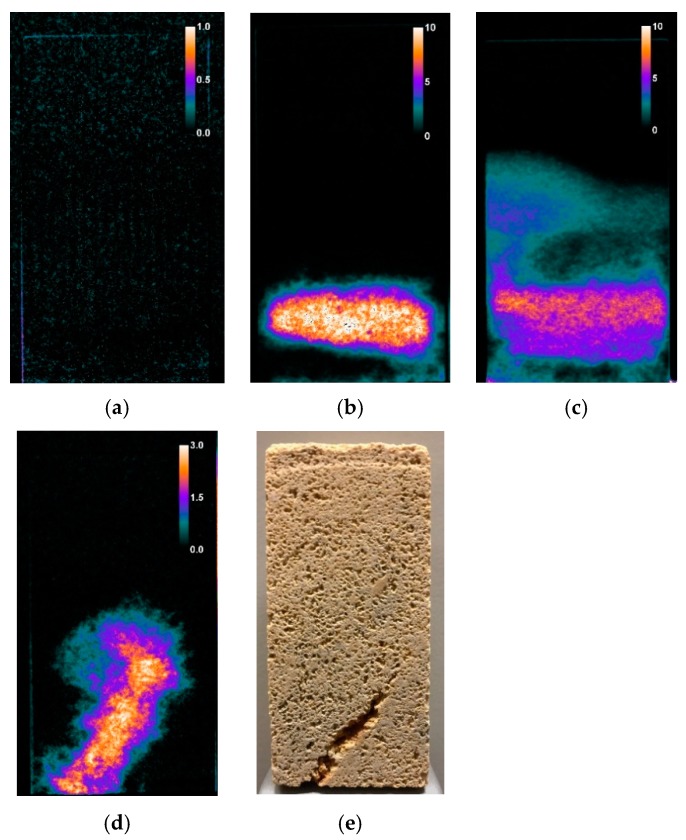
St. Margarethen Limestone radiographs recorded after 30 min of water absorption by capillarity and displaying the water amount in wt. % for specimens treated with NC-25C by (**a**) capillary absorption, (**b**) brushing and (**c**) poultice. A radiograph (**d**) displays the water absorption by capillarity in the presence of a vugular porosity and (**e**) the so-called vugs, which are large cavities in a natural stone, usually exhibiting mineral precipitants on the pore walls.

It was demonstrated that the specimens consolidated with NC-25C and applied by poultice and brushing result, due to the apparent hydrophobicity, in so-called ‘water trapping’ (see effects of this phenomenon in Figure 7b,c). This water trapping occurred in the inner core of the stone specimens, while the lateral surfaces were still mostly hydrophobic with the exception of small passages where capillary forces sucked the water inside the material. This inner core of the stone specimens that was filled with water possibly indicates a polymerisation that was initiated inside the stone or parts of the structure that were not consolidated in the first place. Particularly, Figure 7b suggests that the very bottom of the stone was hydrophobic, and above the water trapping zone the specimen was also hydrophobic, even though it was a highly porous lithotype. The latter radiograph therefore indicates that water was trapped in between the very bottom and first third of the specimen. This represents an unusual phenomenon where hydrophobic and hydrophilic patterns intersect. Trapping of liquid water inside an incompletely polymerised consolidant could jeopardise the silica gel structures, promote the solubility of salts, increase bio-growth or cause spalling of the surface due to differential stresses, all phenomena that potentially lead to harmful effects. Furthermore, neutron imaging was able to show that in the presence of fabric inhomogeneities like the so-called vugs (large pores with calcite crystallization on the pore walls), water uptake was promoted along those structural paths, since these interfaces were most probably not completely covered by the consolidant during the treatment (Figure 7d,e).

The apparent hydrophobicity present due to an incomplete reaction could also be confirmed by the amount of consolidant absorbed and deposited, which was recorded gravimetrically (Figure 5). The mass percentage of the silica gel that formed after curing could be calculated, but only when complete hydrolysis and condensation took place, which in the present study was not the case. Incomplete reactions lead to higher values, as can particularly be seen in the case of samples labelled as over-consolidated (C_NC-25C_Oc). It can be supposed that the over-consolidation of specimens retarded the polymerisation. However, at the present moment it is not clear if the delay of the polymerisation was caused solely by the amount of consolidant applied, or possible pore-clogging caused by repetitive treatments—where gelation has already started but a second application cycle was performed onto the already gelled surfaces. In any case, the results indicate that an important factor for reaction kinetics is the amount of product applied. To further enhance our understanding of this, it would be desirable to study the reaction kinetics of consolidated stone exposed to different environmental conditions in terms of temperature and relative humidity, and with respect to varying amounts of consolidants applied on different substrates.

**Figure 8:**
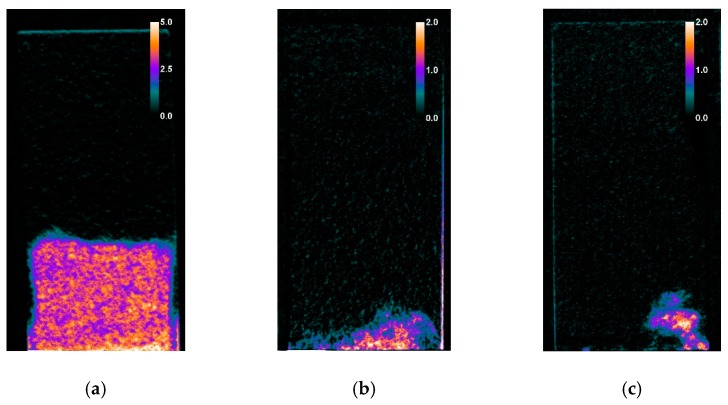
Radiographs of Schlaitdorf Sandstone recorded at 30 min of water absorption by capillarity for specimens treated with NC-25C by (**a**) capillary absorption, (**b**) brushing and (**c**) poultice. The calibration bar displays the water amount in wt. %.

In view of the above observations, it is suggested that the stone fabric plays a decisive role in the polymerisation process, as it can accommodate different amounts of consolidants, which in turn lead to different reaction kinetics. Recent studies by Sena da Fonseca et al. [53] have demonstrated that the silica gel formed in carbonate substrates exhibit poor structural features due to the stone composition, and an in-depth gradient of the polymerisation mechanisms was evident. St. Margarethen Limestone also displays a gradient of polymerisation that is indirectly evident through the amount of water absorbed. Moreover, St. Margarethen Limestone displays anomalous water distributions, evident to a higher degree than can be observed in the more homogeneous silicate variety Schlaitdorf (comparison of the same application techniques on the two studied substrates can be seen in Figure 7b,c and Figure 8b,c). This effect can be ascribed to the different structural features of the studied lithotypes.

The results concerning the Schlaitdorf Sandstone in many regards overlap with the above-mentioned observations concerning St. Margarethen Limestone. With this stone variety, reaction time differences also resulted from different amounts of applied product. For specimens treated with NC-25C, all three application methods allowed the same amount of product to be absorbed by the stone (see Figure 5b and compare C_NC-25C, B_NC-25C and P_NC-25C). This kind of behaviour cannot be observed with St. Margarethen Limestone, and thus can be viewed as a substrate-dependent characteristic of Schlaitdorf Sandstone. The same behaviour for both lithotypes can be seen within the overconsolidated set of specimens, wherein the amount of deposited consolidant does not represent a complete polymerisation, and thus the values of the deposited amount of consolidant are overestimated. Schlaitdorf Sandstone exhibits a slightly faster polymerisation in specimens treated by capillary absorption, which was observed in one out of three studied specimens (Figure 8a). Brushing and poultice caused a more pronounced clogging of the pores, so that water penetrated through small, unconsolidated or already polymerised passages in minor amounts.

The water absorption curves obtained from neutron radiography, as shown in Figure 6, are an average of the entire sample width-depth as a function of the height. The same kind of averaging is present within gravimetrically recorded samples. Imaging proves to be a great added value, because, by qualitative, visual analysis of the radiographs, a more profound discussion of the measured values is possible. Locally, increased amounts of water could be found (compare Figure 6, showing the kinetics of three St. Margarethen specimens treated with brushing, and Figure 7b, showing one radiograph where this kinetic was extracted from). Neutron radiography proved to be a valuable tool to examine anomalous water distributions with a strong gradient of water quantities in the stone, which relates to a gradient in polymerisation.

The optimal curing conditions until complete polymerisation, namely 20 °C and 50% relative humidity for four weeks, can rarely be met in time, which is why treatments with alkoxysilanes must be carefully evaluated, as the outcome of treatment performance is multi-dependent and evolves in time as a function of the substrate, temperature, relative humidity, amount of product and the application method employed. Moreover, the role of sample pre-conditioning in the speed of the chemical reaction is unknown and should be further investigated. That is, laboratory storage before the treatment application should be avoided, because the equilibrium humidity inside the stone structure plays a central role in the initiation of the hydrolysis.

### 3.3. Evaluation of Treatment Application with Nano-SiO_2_ Consolidation on St. Margarethen Limestone and Schlaitdorf Sandstone

Radiographs of samples treated with NC-12C are easier to interpret. As this consolidant is non-reactive, the application technique used, and thus the amount of product deposited, in St. Margarethen Limestone leads to marginal changes with respect to water absorption by capillarity (typical radiographs can be seen in Supplementary Materials in Figure S2). This is a clear advantage of such consolidants when compared to reactive systems like TEOS, at least in the initial stage after the treatment application. As soon as the solvent evaporates, the nano-particles aggregate and strengthen the stone, thus follow-up restoration and conservation activities can be implemented immediately afterwards. The latter is not possible with TEOS until the apparent hydrophobicity vanishes.

**Figure 9:**
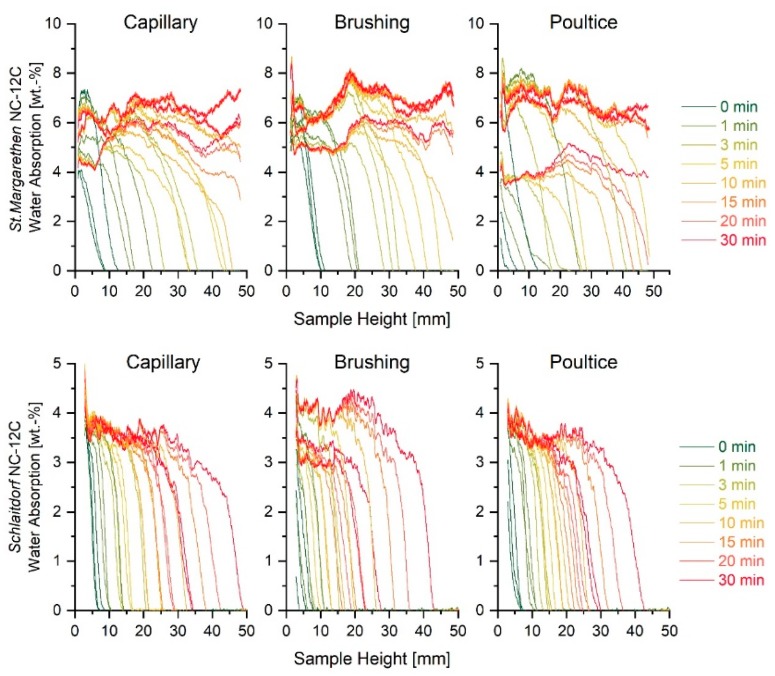
The top graph displays water absorption kinetics reported in weight percentage for St. Margarethen Limestone treated with NC-12C, while the bottom graph displays the kinetics of water absorption by capillarity for Schlaitdorf Sandstone, also treated with NC-12C.

However, it was observed that the set of samples treated with poultice displayed the highest spread of results and anomalous water kinetics, indicating an inhomogeneous product deposition inside St. Margarethen Limestone when compared to the brushing and capillary absorption techniques (Figure 9). Even with the observed differences in kinetics, all three application methods are relatively comparable and display a negligible impact on the water behaviour of consolidated specimens. The nano-particle based systems can be applied in most environmental conditions if proper aftercare is provided (i.e., sun protection, rain coverage, etc.). Moreover, re-application and cyclic treatments are more likely to succeed within such systems. Finally, pre-wetting with the corresponding solvent prior to consolidation seems to have an insignificant impact on the outcome for the given specimen dimensions. Therefore, such pre-treatments for the product NC-12C are safe to perform, as they might reduce the glossiness on the surface. However, as a consequence of pre-wetting, smaller amounts of nanoparticles could be deposited inside the stone, because it dilutes the active content of the consolidant.

Schlaitdorf Sandstone consolidated with NC-12C exhibits similar properties to the carbonate variety St Margarethen Limestone. A comparable amount of product is deposited despite the three different application techniques. The latter is a characteristic attributed to the structure of Schlaitdorf Sandstone, and differs entirely from St. Margarethen Limestone where the amount of adsorbed consolidant depends upon the application technique used (compare the values in Figure 5). The nano-silica consolidation slows down the capillary water absorption, with comparable trends among application techniques (compare the sound set of specimens in Figure 4c with the Figure 9 bottom graphs). However, based on water absorption profiles extracted from neutron imaging, it can be noticed that the water behaviour was more homogenous in specimens treated by capillary absorption. Brushing and poultice is less favoured, as the widespread nature of the results indicates a more inhomogeneous behaviour of water kinetics caused by the treatment application.

### 3.4. Cross-Validation of Neutron Radiography with the Water Absorption Coefficient and Ultrasonic Pulse Velocity

**Figure 10:**
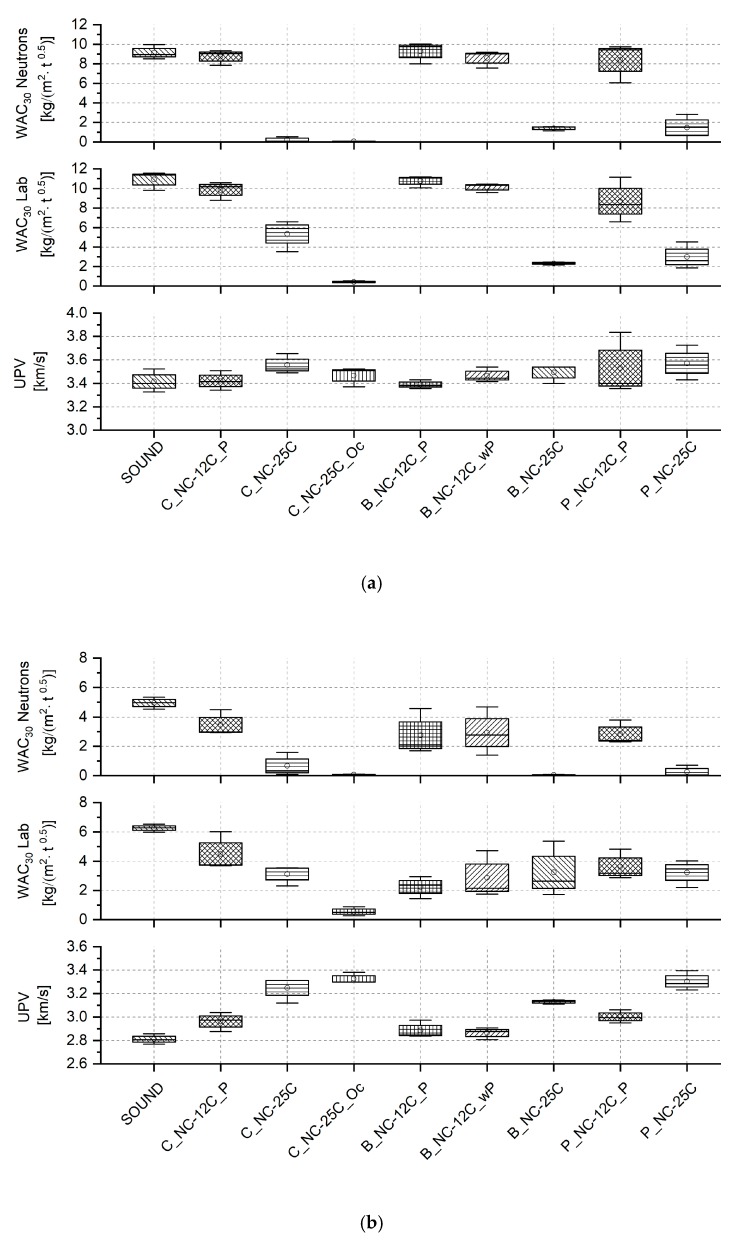
Comparative graph displaying water absorption coefficients reported as [kg/(m^2^·*t*^0.5^)] after 30 min for (**a**) St. Margarethen Limestone and (**b**) Schlaitdorf Sandstone, calculated from (**top**) neutron radiography and (**middle**) gravimetrically determined laboratory studies, and compared to values of (**bottom**) ultrasonic pulse velocity reported in [km/s]. Corresponding numerical values of the graphical representation can be extracted from Supplementary Materials, Table S2. Moreover, all water absorption curves extracted from neutron radiographs can be seen in Supplementary Materials, Figure S3 (St. Margarethen Limestone) and Figure S4 (Schlaitdorf Sandstone).

To cross validate the water absorption by capillarity, monitored by neutron radiography, the WAC calculated from the laboratory mass increase, as well as UPV, were determined on the same specimens. The comparative values displayed in Figure 10a,b show that there is a clear trend between all tests employed to study alterations caused by the treatment application. When the WAC increased, UPV decreased accordingly. As already mentioned above, the slight difference between absolute values reported for the WAC gained from neutron radiography and laboratory-based gravimetrical results can be explained through averaging when image analysis is used. The impact of the advanced polymerisation is visible in the WAC values, based on gravimetrically determined laboratory studies. Those values evolved over time, thus suggesting a more advanced chemical reaction, when compared to the WAC obtained through neutron radiography. The latter effect is evident when comparing calculations from neutrons and laboratory studies of NC-12C with NC-25C values. This comes as no surprise, as these tests were performed eight weeks after consolidation and the specimens had already been in contact with water through previous water absorption tests when the neutron radiographs were acquired.

One of the main findings is that, in order to study petrophysical changes caused by treatment application by means of neutron radiography, sound samples proved to be convenient. However, the use of sound stone specimens to evaluate the efficacy of a consolidant should be discarded. The reason for this is evident in the comparative graph, because the increase in UPV is minor and can be misinterpreted. Such a slight increase does not rest solely on the treatment’s performance, but rather on the conditions of the substrate under study. The hypothesis is that when a sound stone is consolidated, the product will precipitate inside the pores but might not influence the grain contacts, as these will be intact in a freshly quarried specimen. Thus, the fastest way for a sound wave to propagate through the sample might still be the same as before the consolidation, since the grain contacts were not affected. Therefore, if mechanical strength gain is the subject of study, it is recommended to artificially age and subsequently consolidate specimens and compare those two states with each other. Only in this way can the potential of the consolidant’s efficiency, which is defined as the restoration of the mechanical strength of the material, be obtained.

Regarding Schlaitdorf Sandstone, the values of UPV are higher in magnitude when compared to St. Margarethen Limestone. The reason for such a behaviour lies in its micro-texture, which was already reported in a previous study [40]. The consolidation of the clayey matrix, which is homogeneously deposited between the grains of Schlaitdorf Sandstone, lead to a faster propagation of the sound wave, and thus higher values after consolidation. The consolidant NC-25C had a higher impact on the velocity, indicating a higher efficiency than the nano-particle based NC-12C. Moreover, an increase in the amount of applied consolidant resulted in an increase in UPV; thus, higher efficiency is the result. The latter also demonstrates that the efficiency and compatibility of treatments will depend on the amount of applied consolidant.

**Figure 11:**
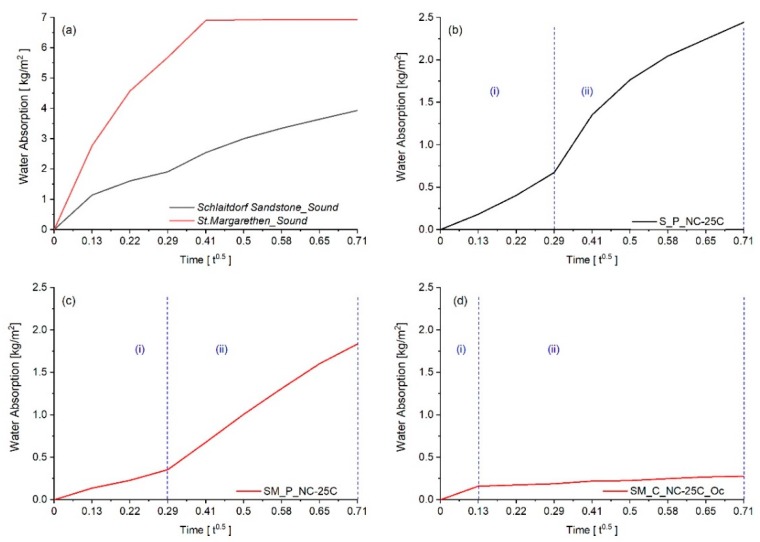
Examples of water absorption curves after 30 min (here reported as the square root of time in hours), extracted from gravimetrical monitoring. Graph (**a**) represents absorption curves from sound stone specimens, (**b**) Schlaitdorf Sandstone, (**c**) St. Margarethen Limestone where the consolidant NC-25C was applied by poultice and (**d**) a specimen of St. Margarethen Limestone representing the over-consolidation attained by a repetitive capillary absorption of NC-25C.

The water absorption curves are more complicated to interpret when the stone specimens are consolidated, especially when reactive treatments are present. Figure 11b,c displays two curves, depicted as part (i) and (ii) in the graphs, which correspond to changes in slope and thus in water absorption kinetics. For both cases, it can be observed that the water absorption increased steadily, and after five minutes a speed increase of the capillary suction took place. The accelerated water absorption rate, represented as part (ii) where the slope increases, represents a phenomenon where a greater pore volume contributes to the suction and increases the slope. Figure 11d represents a water absorption curve of a hydrophobic specimen, where water is initially absorbed on the surface (measurement at 1 min) and diffuses gradually with time. In the latter case, the magnitude of absorbed water per surface area displays the water repellence or hindrance of the stone specimen. The evolution of water absorption kinetics as the polymerisation proceeds should be investigated more in depth by means of neutron radiography and additional laboratory water-based methods.

## 4. Conclusions

Weathering is known to cause a loss of grain cohesion, and thus a reduction of mechanical strength in monumental stone. To regain the lost cohesion, stone consolidants are frequently applied. In the present study, neutron radiography was used to monitor water absorption by capillarity, and to analyse possible alterations caused by artificial ageing of stone and three treatment applications, applied on two different substrates and treated with two consolidants.

The results show that artificially aged or heat-treated stone specimens must not be used when physical changes are studied, as the impact seen by neutron radiography is limited. On the contrary, when studying mechanical strength changes, artificial ageing is a prerequisite in order to study the efficiency of stone consolidants, as findings with UPV demonstrate. When grain contacts are not affected, as is the case in a sound material, the consolidation efficiency will yield underestimated values, which are not representative of the capability of the consolidant to strengthen the substrate.

Concerning the evaluation of treatment applications with stone consolidants by means of neutron radiography, the results reveal that the amount of product applied, and therefore the application technique used, is relevant when using a reactive system like NC-25C—the nano-titania doped tetraethyl-orthosilicate. On the contrary, the nano-silica-based consolidant NC-12C shows comparable results regarding water absorption kinetics regardless of the substrate or treatment application used. For the reactive system NC-25C, different amounts of product applied result in different speeds of chemical reaction, which makes the nano-based consolidant NC-12C more favourable for on-site work. However, the advantage of NC-25C is the higher mechanical strength when compared to NC-12C, as observed by UPV. The differences in the degree of polymerisation are particularly visible on the carbonate variety St. Margarethen Limestone, ranging from water repellence to anomalous water kinetics ascribed to partial polymerisation and initiated in the inner core of the stone specimens. In the latter case, when NC-25C was applied by brushing and poultice, pore clogging in the sub surface area and water trapping, as well as anomalous capillary absorption, were recorded.

Neutron radiography proved to be an excellent tool for dynamic observations of the inner pathways of adsorbed water in a consolidated substrate. It gave insights on the effects of stone texture and structure, the preferential pathways of water being absorbed and distributed throughout the material, which is relevant in determining the final behaviour that cannot be revealed by simple lab-based water absorption tests. While WAC is simply averaging all phenomena and quantities, neutron radiography allowed to see localised quantities, the degree of spreading of the same and the kinetics of those processes. Without neutron imaging, the present study would not be able to observe that the amount of absorbed water in aged samples was higher, but that the speed in some lithotypes and localities slowed down. Furthermore, it would have been impossible to observe that the polymerisation was initiated inside the stone while the outer surfaces were still mostly hydrophobic. Moreover, the vugular porosity that is texturally different from the surrounding structure of the stone indeed represented a preferential pathway for the water in a consolidated stone. Additionally, if the specimens seemed hydrophobic, there was a small passage where the water penetrated into the depth of the stone and did not just stay in the surface zone. All those conclusions would not have been possible without the use of neutron imaging.

The present study focused on two porous lithotypes, but in order to draw wider conclusions relevant to the field and to possibly establish guidelines for treatment applications, the same experimental set-up should be employed on additional lithotypes and with additional consolidating materials. Variation of the amount of consolidant applied, the studied time span after treatment application and different curing conditions should be considered for further studies. Moreover, other application techniques should also be analysed, as well as lithotypes with different natural and artificial decay patterns.

## Figures and Tables

**Table 1:** Labelling for the studied conditions: sound and aged stone specimens as reference conditions, consolidated specimens with either NC-12C or NC-25C applied by different application techniques: capillary absorption, brushing and poultice for both lithotypes.

Reference Specimens	Consolidated Specimens		
	Capillary	Brushing	Poultice
Sound	C_NC-12C_P ^1^	B_NC-12C_P ^1^	P_NC-12C_P ^1^
Aged	C_NC-25C	B_NC-12C_wP ^3^	P_NC-25C
	C_NC-25C_Oc ^2^	B_NC-25C	

^1^ Pre-wetting. ^2^ Overconsolidated. ^3^ Without pre-wetting.

**Table 2:** Difference between water absorption coefficients calculated after 30 min (*t*) and 5 min (*t*) for sound and aged St. Margarethen Limestone and Schlaitdorf Sandstone, reported as [kg/(m^2^ *t*^0.5^)].

**St. Margarethen Limestone**								
**Calculation**	**Neutron Radiography**				**Gravimetrical Increase**			
**Condition**	**Sound**		**Aged**		**Sound**		**Aged**	
WAC [min]	WAC_30_	WAC_5_	WAC_30_	WAC_5_	WAC_30_	WAC_5_	WAC_30_	WAC_5_
Coefficient	9.15	19.92	11.36	22.28	10.92	20.94	12.83	23.64
SD.N.	±0.61	±1.41	±0.87	±1.76	±0.80	±1.01	±0.23	±0.94
**Schlaitdorf Sandstone**								
**Calculation**	**Neutron Radiography**				**Gravimetrical Increase**			
**Condition**	**Sound**		**Aged**		**Sound**		**Aged**	
WAC [min]	WAC_30_	WAC_5_	WAC_30_	WAC_5_	WAC_30_	WAC_5_	WAC_30_	WAC_5_
Coefficient	4.94	5.70	5.51	6.47	6.26	6.81	5.75	6.75
SD.N.	±0.34	±0.56	±0.41	±0.47	±0.22	±0.41	±0.18	±0.24

## Supplementary Materials

The following are available online at https://www.mdpi.com/2079-4991/9/4/635/s1, Figure S1. The upper plot displays the temperature (°C) and relative humidity (%), recorded once per day in the city centre of Pisa, Italy during May 2018. The bottom plot shows the monitored weight of consolidated St. Margarethen Limestone and Schlaitdorf Sandstone stone specimens. An average of three specimens is shown for each treatment and lithotype. The labelling C_NC-25C stands for treatment application by capillary absorption with nano-titania modified tetraethyl-orthosilicate, while P_NC-12C_P stands for treatment by poultice, pre-wetted by ethanol prior to treatment application. Table S1. Water absorption coefficient [kg/(m^2^·*t*^0.5^)] after 30 min calculated from neutron imaging and gravimetrical laboratory analysis as well as ultrasonic pulse velocity [km/s] before (sound) and after artificial ageing (aged) for St. Margarethen Limestone and Schlaitdorf Sandstone. Average data of three stone specimens. Figure S2. Normalised images of sound (top) and consolidated (bottom) St. Margarethen Limestone with the nano-silica consolidant (NC-12C) applied by brushing. Table S2. Comparative graph displaying water absorption coefficients after 30 min [kg/(m^2^·*t*^0.5^)] calculated from neutron radiography and laboratory tests by gravimetrical means and compared with ultrasonic pulse velocity [km/s] for St. Margarethen Limestone and Schlaitdorf Sandstone. Figure S3. Water absorption (wt. %) extracted from neutron radiography scans on three specimens per lithotype and condition (sound, aged, consolidated). Figure S4. Water absorption (wt. %) extracted from neutron radiography scans on three specimens per lithotype and condition (sound, aged, consolidated).
