# Supplementary material for: Neutron Radiography Study of Laboratory Ageing and Treatment Applications with Stone Consolidants
Source: Nanomaterials (Basel). 2019 Apr 19;9(4):635. doi: 10.3390/nano9040635 (PMC6523362; doi:10.3390/nano9040635)

nanomaterials

MDPI

Supplementary Materials

Neutron Radiography Study of Laboratory Ageing and Treatment Applications with Stone Consolidants

Matea Ban [1,*], Tim De Kock [2], Frédéric Ott [3], Germana Barone [4], Andreas Rohatsch [1] and Simona Raneri [5]

[1] Institute of Geotechnics, Research Centre of Engineering Geology, Vienna University of Technology, 1040 Vienna, Austria; andreas.rohatsch@tuwien.ac.at
[2] Department of Geology, Ghent University, 9000 Ghent, Belgium; Tim.DeKock@UGent.be
[3] Laboratoire Léon Brillouin, Université Paris-Saclay, Centre d'Etudes de Saclay, 91191 Gif sur Yvette CEDEX, France; Frederic.Ott@cea.fr
[4] Department of Biological, Geological and Environmental Sciences, University of Catania, 95129 Catania, Italy; gbarone@unict.it
[5] Department of Earth Sciences, University of Pisa, 56126 Pisa, Italy; simona.raneri@unipi.it
* Correspondence: matea.ban@tuwien.ac.at; Tel.: +43-158-801-20318

Supplementary Graph for the Section 2.4. Consolidation of Stone: Treatment Procedure and Curing

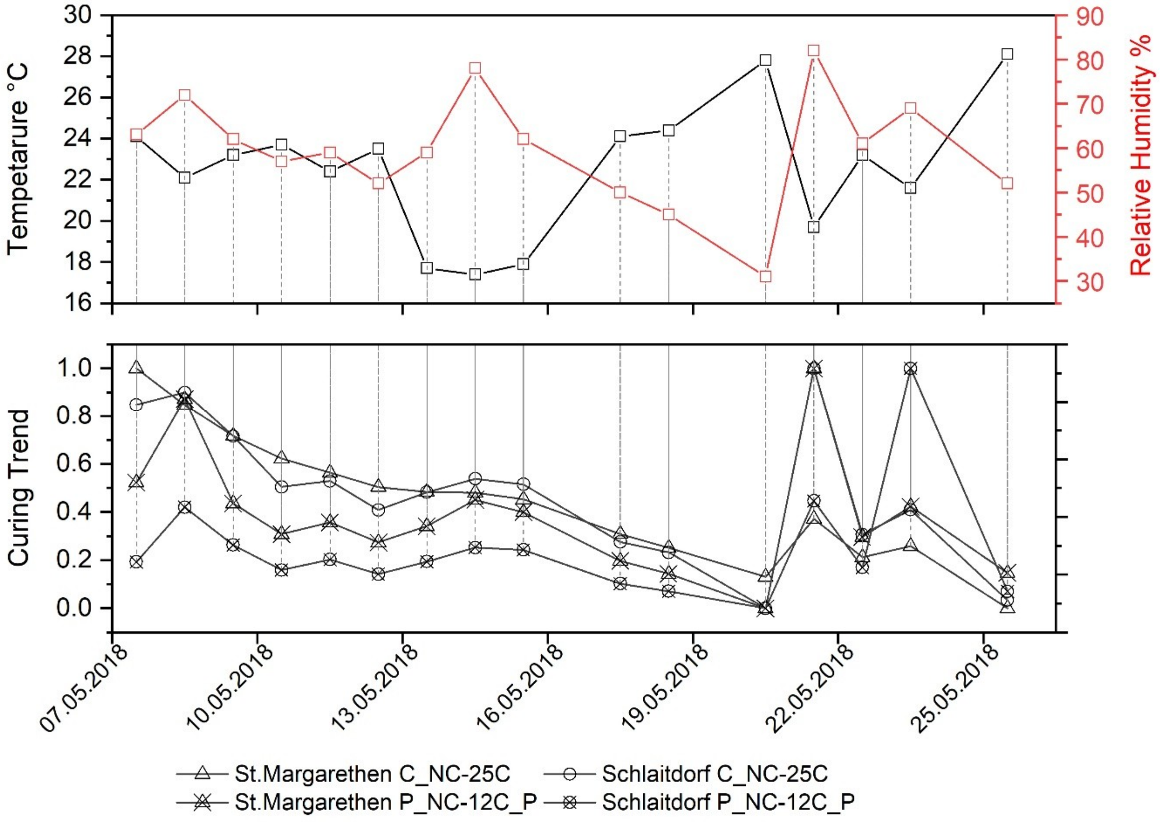

Figure S1. The upper plot displays the temperature (°C) and relative humidity (%), recorded once per day in the city centre of Pisa, Italy during May 2018. The bottom plot shows the monitored weight of consolidated *St. Margarethen Limestone* and *Schlaitdorf Sandstone* stone specimens. An average of three specimens is shown for each treatment and lithotype. The labelling C_NC-25C stands for treatment application by capillary absorption with nano-titania modified tetraethyl-orthosilicate, while P_NC-12C_P stands for treatment by poultice, prewetted by ethanol prior to treatment application.

Supplementary Table for the Section 3.1. Evaluation of the Artificial Ageing

Table S1. Water absorption coefficient [kg/(m$^2\cdot$ t$^{0.5}$)] after 30 minutes calculated from neutron imaging and gravimetrical laboratory analysis as well as ultrasonic pulse velocity [km/s] before (**sound**) and after artificial ageing (**aged**) for *St. Margarethen Limestone* and *Schlaitdorf Sandstone*. Average data of three stone specimens.

St. Margarethen Limestone						
	WAC Neutron Radiography		WAC Laboratory		Ultrasonic Pulse Velocity	
	Sound	Aged	Sound	Aged	Sound	Aged
WAC	9.15	11.36	10.92	12.83	3.42	1.7
SD.N.	±0.61	±0.87	±0.80	±0.23	±0.08	±0.03
Schlaitdorf Sandstone						
	WAC Neutron Radiography		WAC Laboratory		Ultrasonic Pulse Velocity	
	Sound	Aged	Sound	Aged	Sound	Aged
WAC	4.94	5.51	5.75	6.26	2.81	1.74
SD.N.	±0.34	±0.41	±0.18	±0.22	±0.04	±0.01

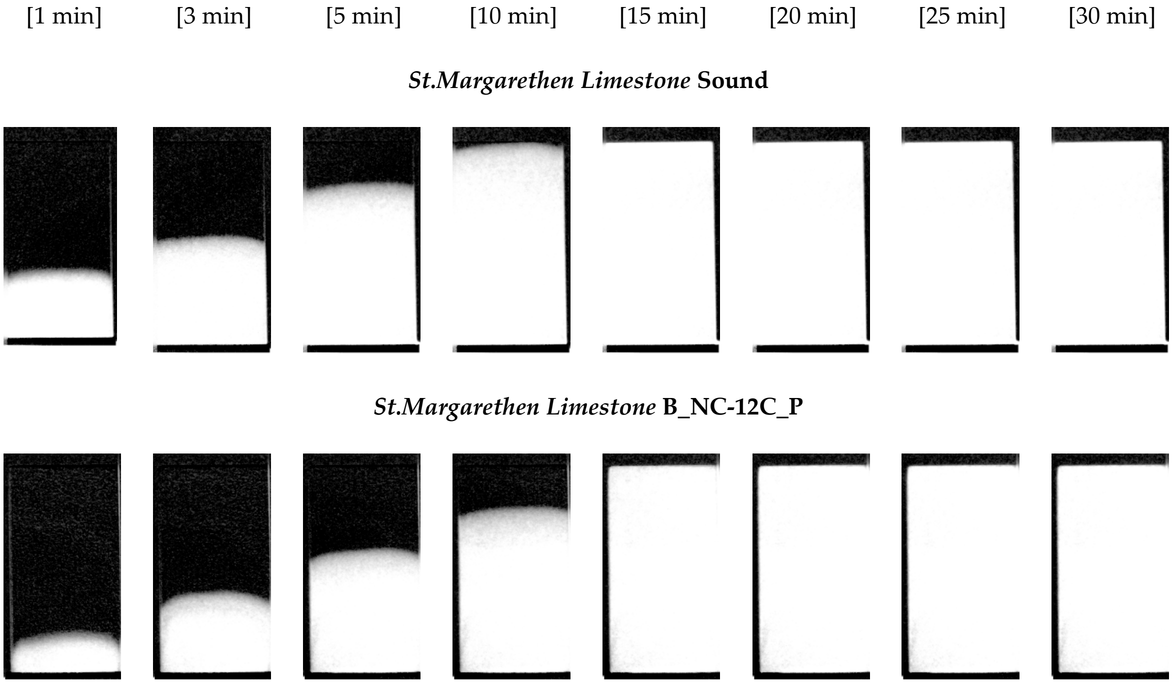

Figure S2. Normalised images of sound (**top**) and consolidated (**bottom**) *St. Margarethen Limestone* with the nano-silica consolidant (NC-12C) applied by brushing.

Supplementary Table for the Section 3.4. Cross-Validation of Neutron Radiography with Water Absorption Coefficient and Ultrasonic Pulse Velocity

Table S2. Comparative graph displaying water absorption coefficients after 30 minutes [kg/($m^2 \cdot t^{0.5}$)] calculated from neutron radiography and laboratory tests by gravimetrical means and compared with ultrasonic pulse velocity [km/s] for *St. Margarethen Limestone* and *Schlaitdorf Sandstone*.

St. Margarethen Limestone									
WAC Neutron Radiography									
		Capillary			Brushing			Poultice	
	SOUND	C_NC-12C_P	C_NC-25C	C_NC-25C_Oc	B_NC-12C_P	B_NC-12C_wP	B_NC-25C	P_NC-12C_P	P_NC-25C
WAC	9.15	8.74	0.23	0.06	9.26	8.59	1.40	8.41	1.45
SD.N.	±0.61	±0.64	±0.21	±0.00	±0.89	±0.72	±0.20	±1.66	±1.14
WAC Laboratory									
		Capillary			Brushing			Poultice	
	SOUND	C_NC-12C_P	C_NC-25C	C_NC-25C_Oc	B_NC-12C_P	B_NC-12C_wP	B_NC-25C	P_NC-12C_P	P_NC-25C
WAC	10.92	9.85	5.34	0.43	10.78	10.12	2.33	8.69	2.99
SD.N.	±0.80	±0.78	±1.31	±0.09	±0.50	±0.38	±0.12	±1.87	±1.12
Ultrasonic Pulse Velocity									
		Capillary			Brushing			Poultice	
	SOUND	C_NC-12C_P	C_NC-25C	C_NC-25C_Oc	B_NC-12C_P	B_NC-12C_wP	B_NC-25C	P_NC-12C_P	P_NC-25C
WAC	3.42	3.42	3.56	3.47	3.39	3.47	3.49	3.53	3.57
SD.N.	±0.08	±0.07	±0.07	±0.07	±0.03	±0.05	±0.07	±0.22	±0.12
Schlaitdorf Sandstone									
WAC Neutron Radiography									
		Capillary			Brushing			Poultice	
	SOUND	C_NC-12C_P	C_NC-25C	C_NC-25C_Oc	B_NC-12C_P	B_NC-12C_wP	B_NC-25C	P_NC-12C_P	P_NC-25C
WAC	4.94	3.47	0.66	0.07	2.76	2.94	0.05	2.83	0.26
SD.N.	±0.34	±0.72	±0.66	±0.03	±1.28	±1.34	±0.03	±0.68	±0.32
WAC Laboratory									
		Capillary			Brushing			Poultice	
	SOUND	C_NC-12C_P	C_NC-25C	C_NC-25C_Oc	B_NC-12C_P	B_NC-12C_wP	B_NC-25C	P_NC-12C_P	P_NC-25C
WAC	5.75	4.49	3.12	0.56	2.24	2.87	3.24	3.63	3.23
SD.N.	±0.18	±1.08	±0.58	±0.25	±0.62	±1.32	±1.55	±0.85	±0.76
Ultrasonic Pulse Velocity									
		Capillary			Brushing			Poultice	
	SOUND	C_NC-12C_P	C_NC-25C	C_NC-25C_Oc	B_NC-12C_P	B_NC-12C_wP	B_NC-25C	P_NC-12C_P	P_NC-25C
WAC	2.81	2.96	3.25	3.33	2.89	2.86	3.13	3.0	3.3
SD.N.	±0.04	±0.07	±0.09	±0.04	±0.06	±0.04	±0.01	±0.05	±0.07

St. Margarethen Limestone

Figure S3. Water absorption (wt-%) extracted from neutron radiography scans on three specimens per lithotype and condition (sound, aged, consolidated).

St. Margarethen_Sound_Sample 1

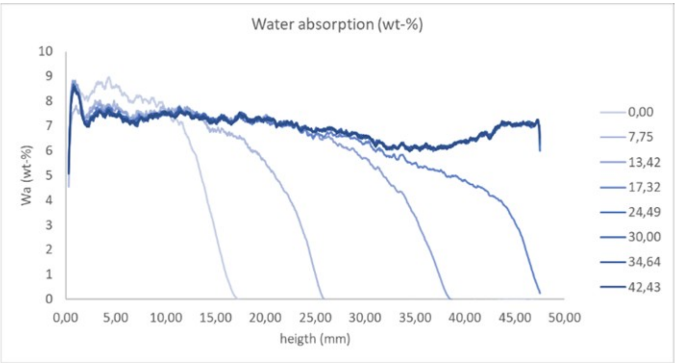

St. Margarethen_Sound _Sample 2

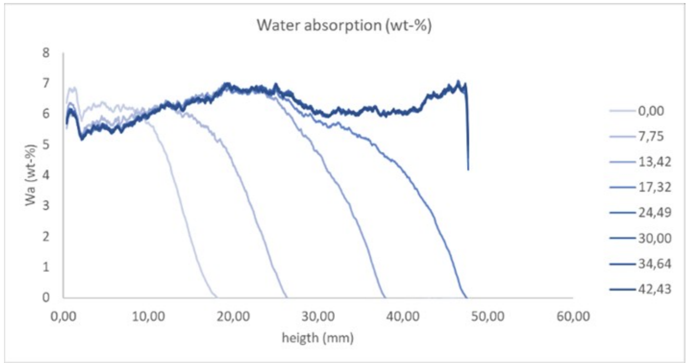

St. Margarethen_Sound _Sample 3

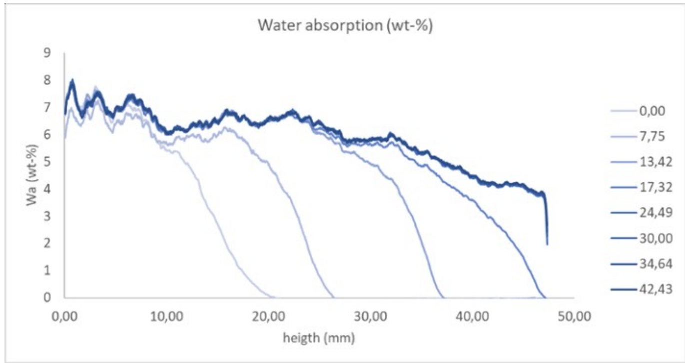

St. Margarethen_Aged_Sample 1

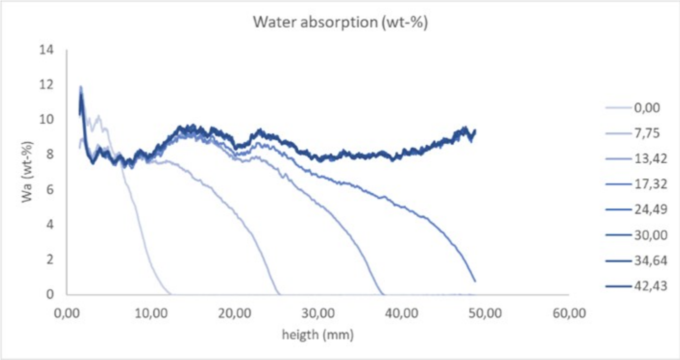

St. Margarethen_Aged _Sample 2

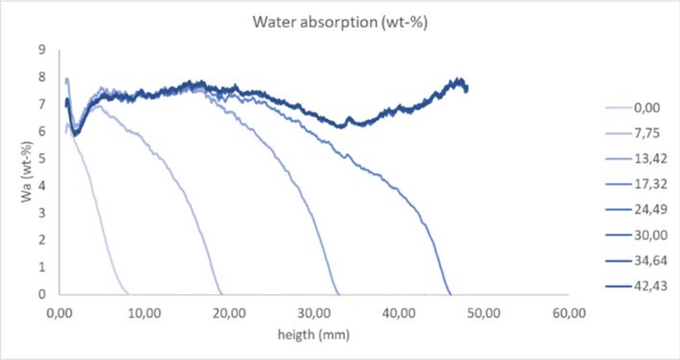

St. Margarethen_Aged_Sample 3

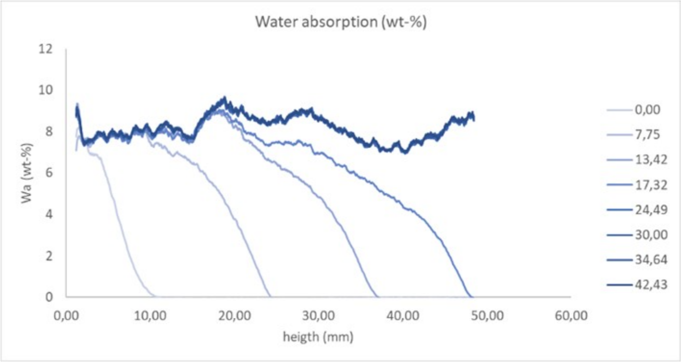

St. Margarethen_C_NC-12C_P_Sample 1

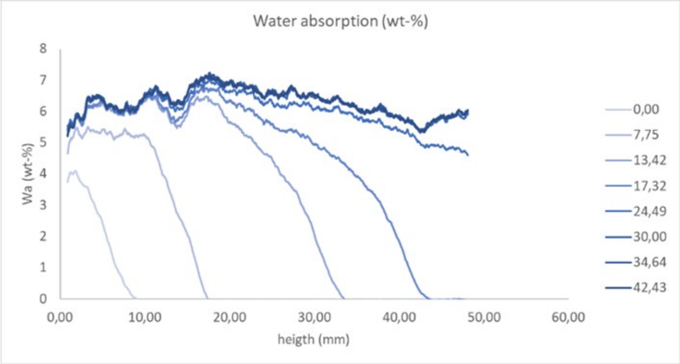

St. Margarethen_ C_NC-12C_P_Sample 2

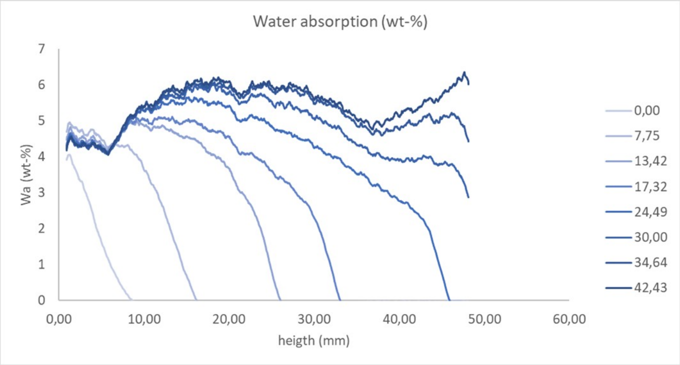

St. Margarethen_C_NC-12C_P_Sample 3

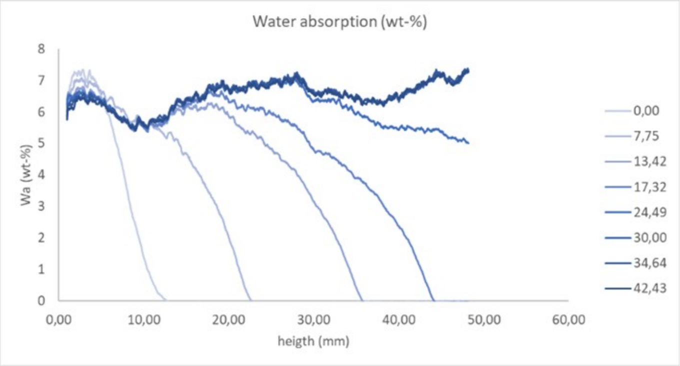

St. Margarethen_C_NC-25C_Sample 1

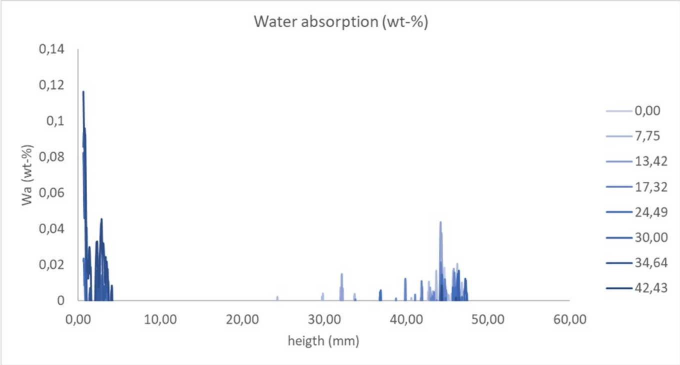

St. Margarethen_C_NC-25C_Sample 2

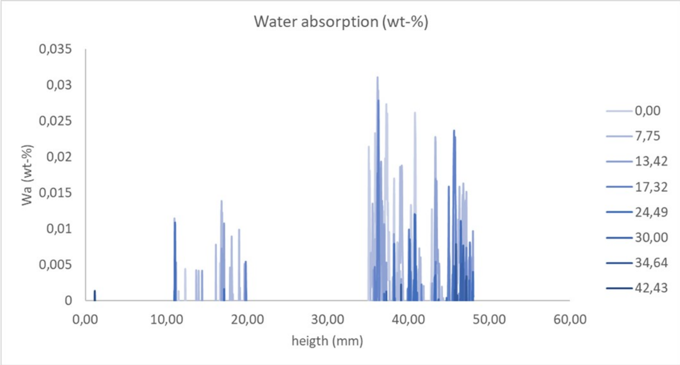

St. Margarethen_C_NC-25C_Sample 3

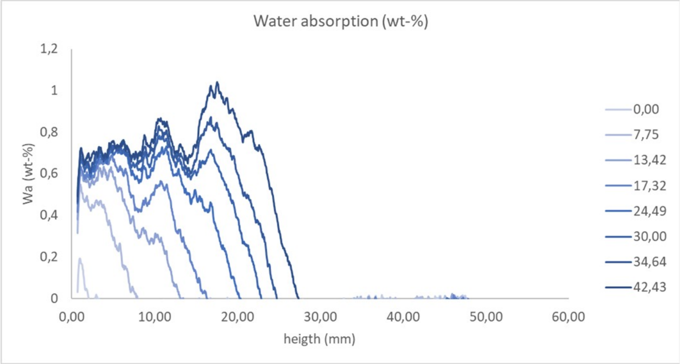

St. Margarethen_C_NC-25C_Oc_Sample 1

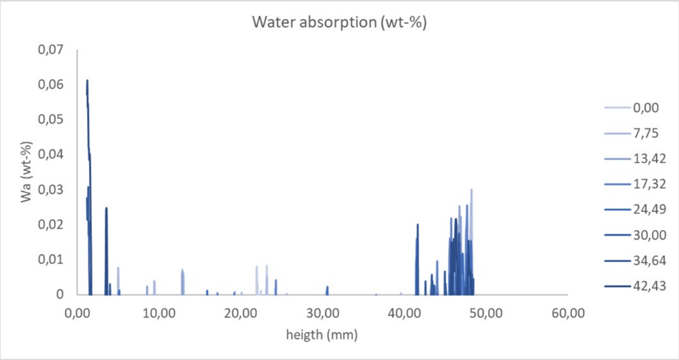

St. Margarethen_C_NC-25C_Oc_Sample 2

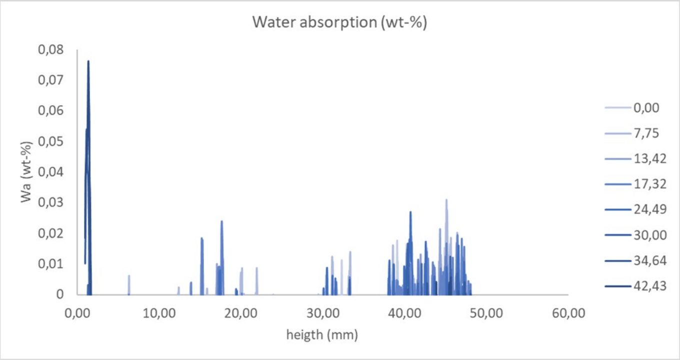

St. Margarethen_C_NC-25C_Oc_Sample 3

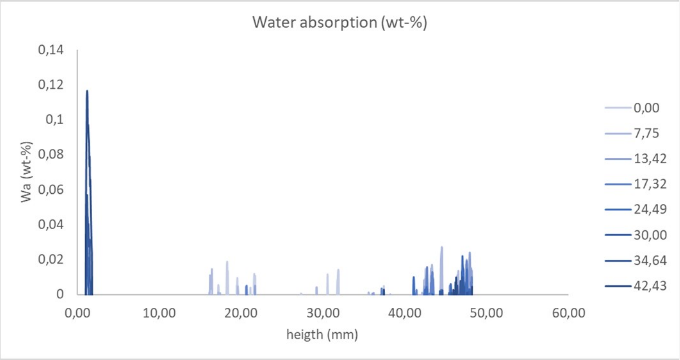

St. Margarethen_B_NC-12C_P_Sample 1

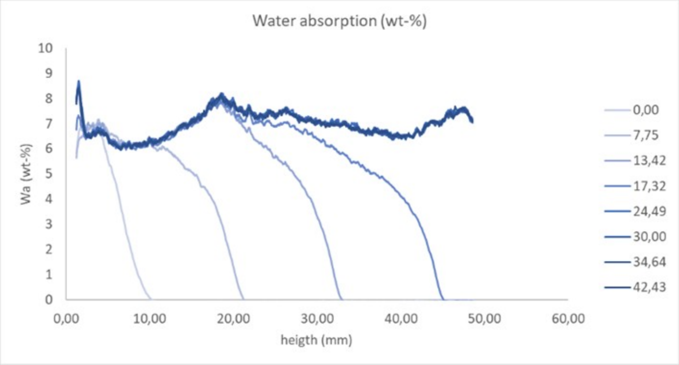

St. Margarethen_B_NC-12C_P_Sample 2

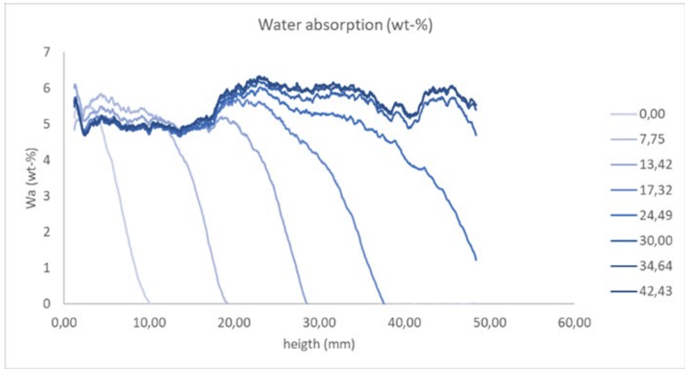

St. Margarethen_B_NC-12C_P_Sample 3

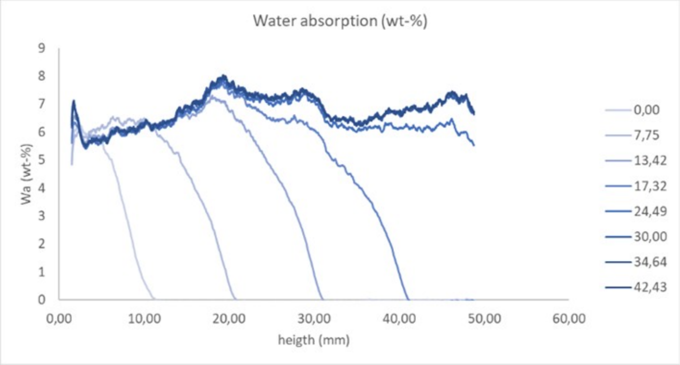

St. Margarethen_B_NC-12C_wP_Sample 1

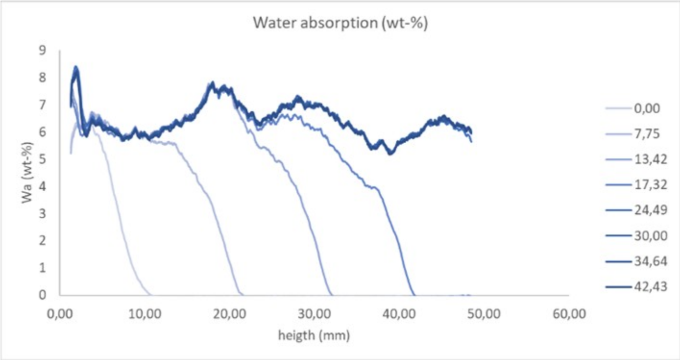

St. Margarethen_B_NC-12C_wP _Sample 2

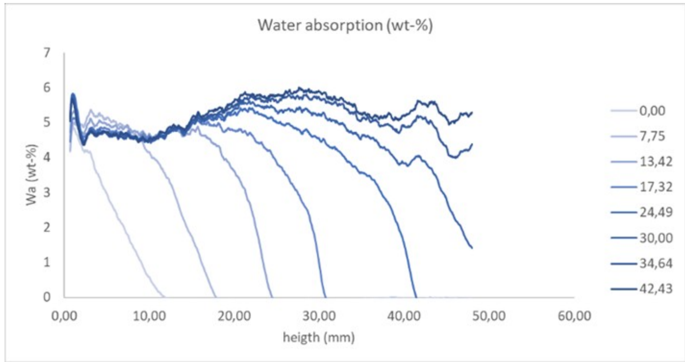

St. Margarethen_B_NC-12C_wP_Sample 3

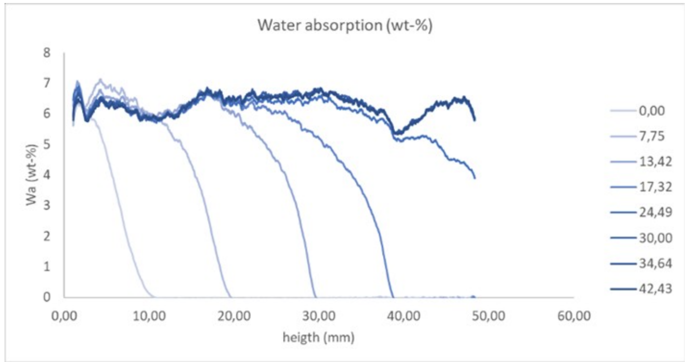

St. Margarethen_B_NC-25C_Sample 1

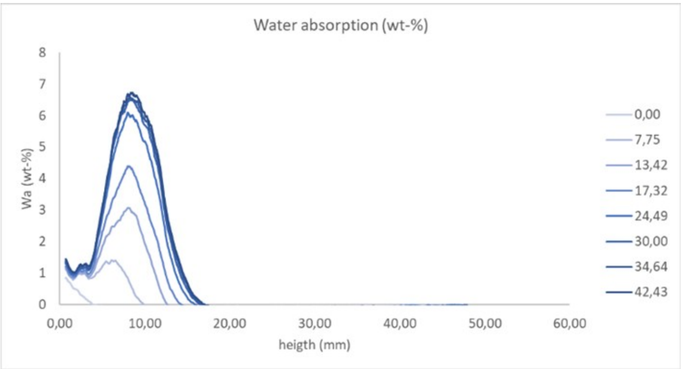

St. Margarethen_B_NC-25C_Sample 2

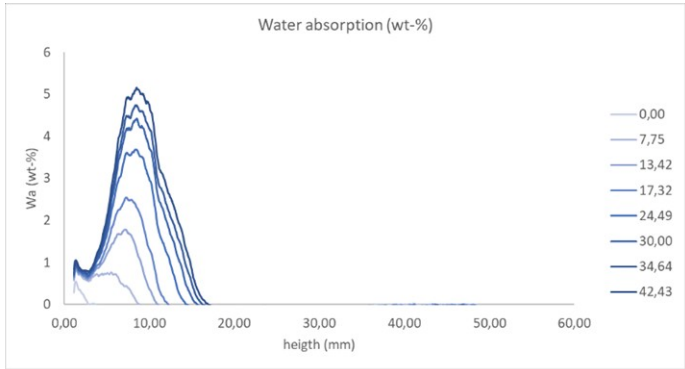

St. Margarethen_B_NC-25C_Sample 3

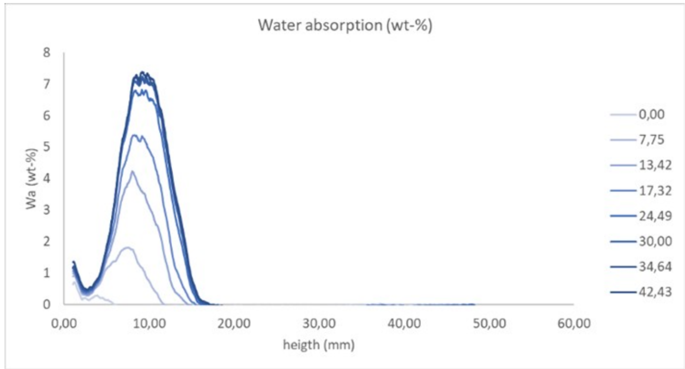

St. Margarethen_P_NC-12C_P_Sample 1

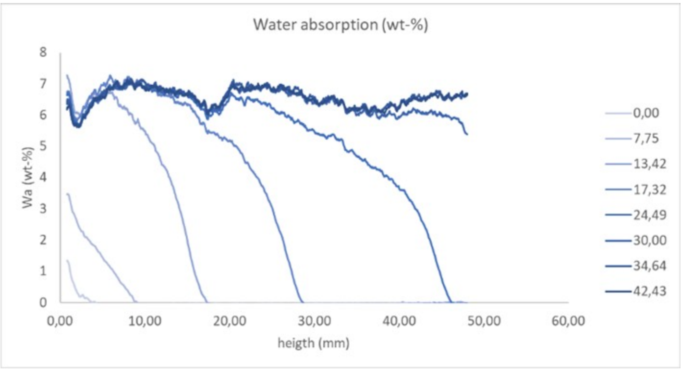

St. Margarethen_P_NC-12C_P _Sample 2

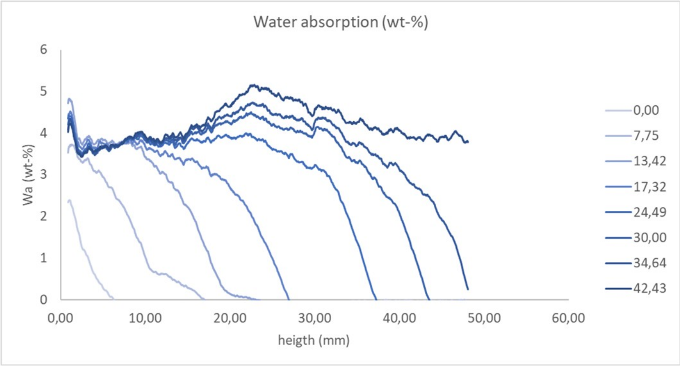

St. Margarethen_P_NC-12C_P_Sample 3

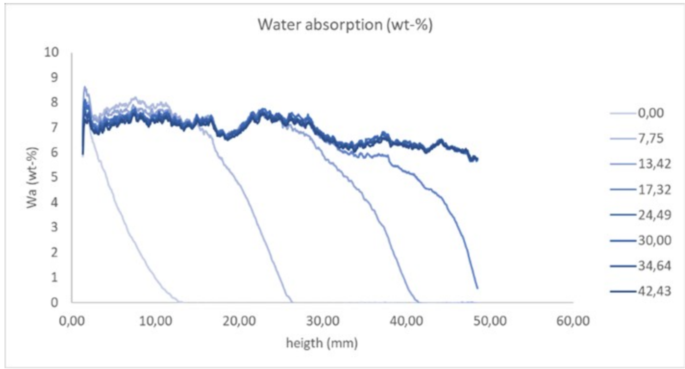

St. Margarethen_P_NC-25C_Sample 1

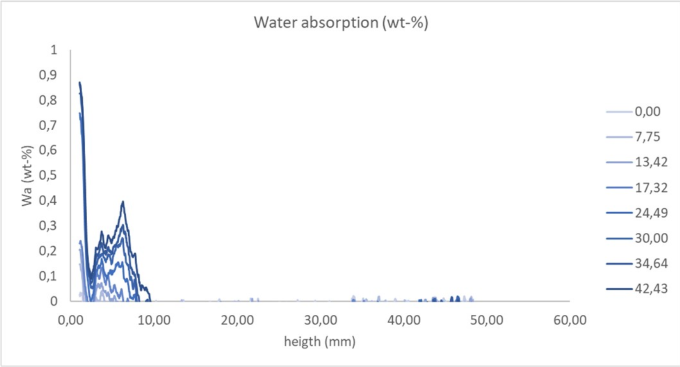

St. Margarethen_P_NC-25C_Sample 2

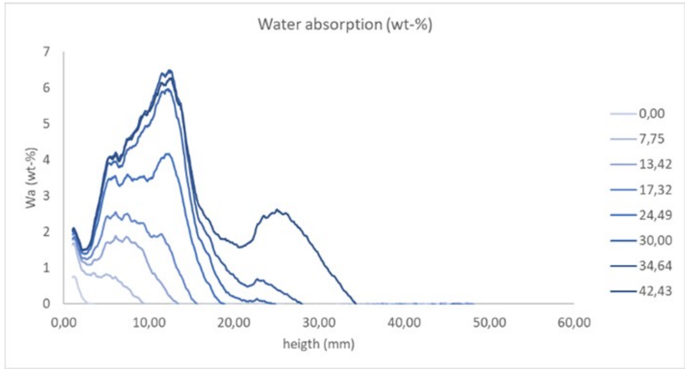

St. Margarethen_P_NC-25C_Sample 3

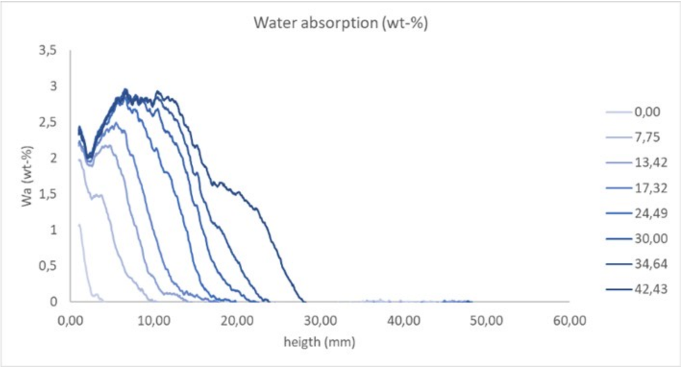

Schlaitdorf Sandstone

Figure S4. Water absorption (wt-%) extracted from neutron radiography scans on three specimens per lithotype and condition (sound, aged, consolidated).

Schlaitdorf Sandstone_Sound_Sample 1

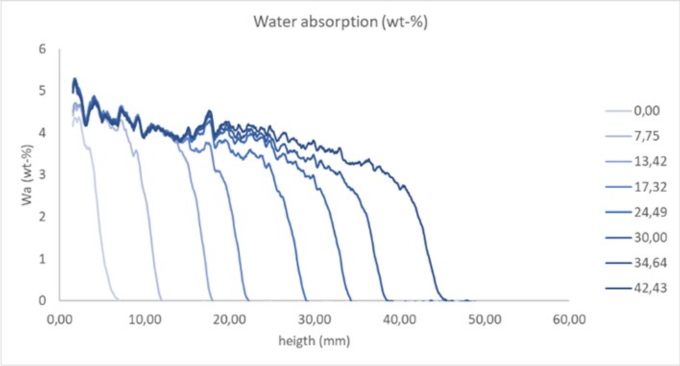

Schlaitdorf Sandstone _Sound_Sample 2

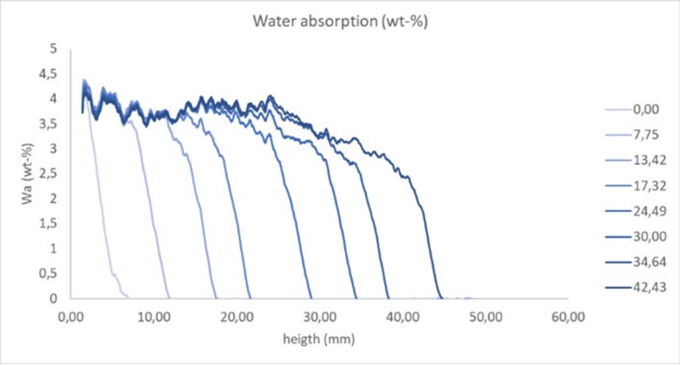

Schlaitdorf Sandstone_Sound_Sample 3

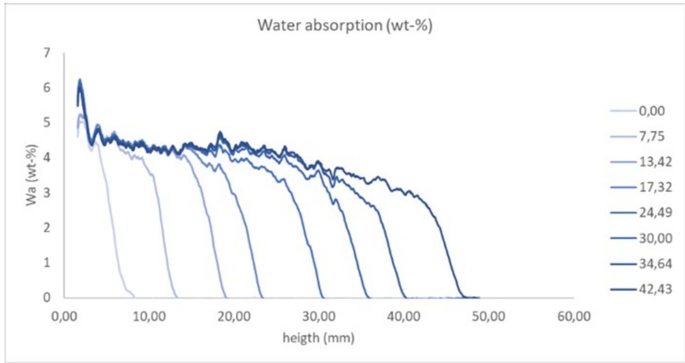

Schlaitdorf Sandstone_Aged_Sample 1

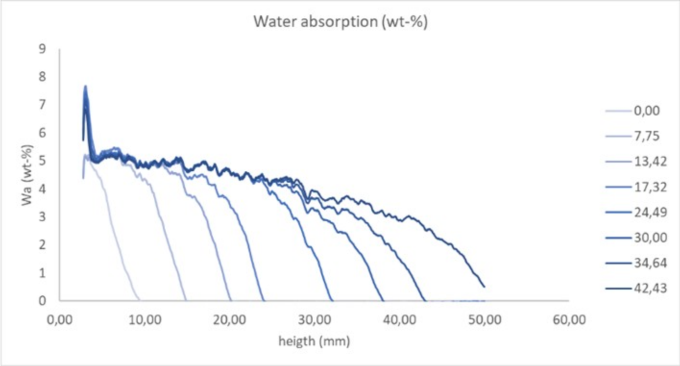

Schlaitdorf Sandstone_Aged_Sample 2

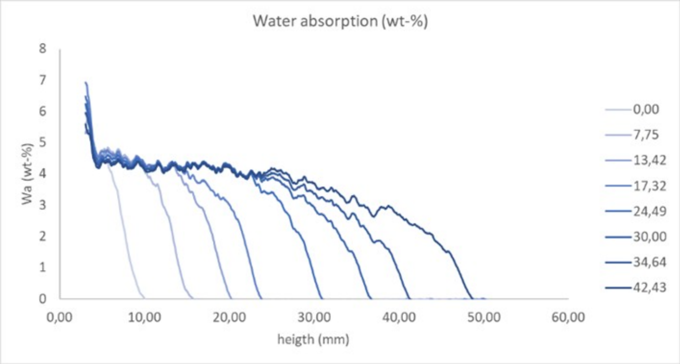

Schlaitdorf Sandstone_Aged_Sample 3

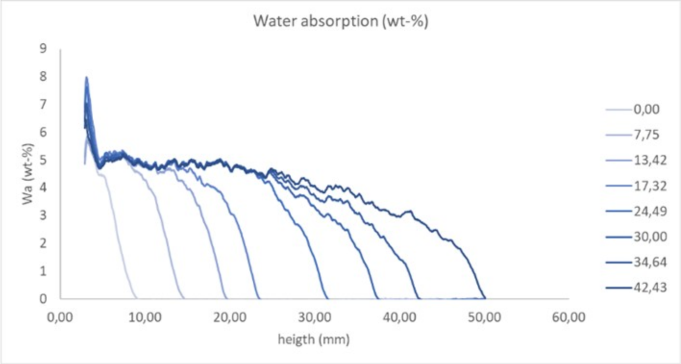

Schlaitdorf Sandstone_C_NC-12C_P_Sample 1

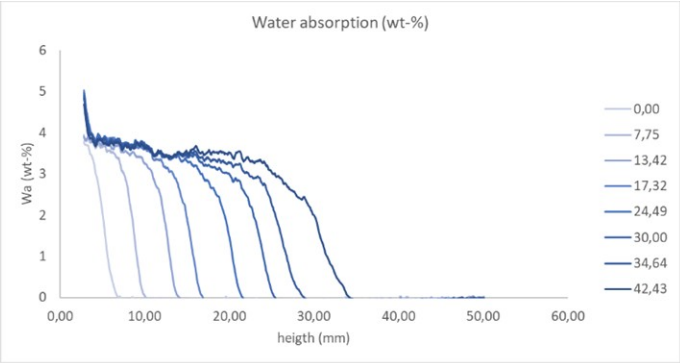

Schlaitdorf Sandstone_C_NC-12C_P_Sample 2

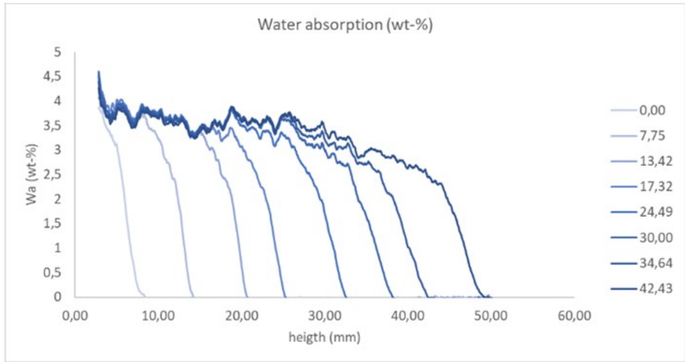

Schlaitdorf Sandstone_C_NC-12C_P_Sample 3

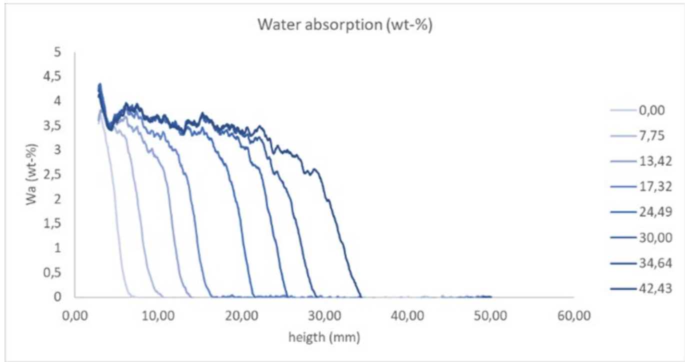

Schlaitdorf Sandstone_C_NC-25C_Sample 1

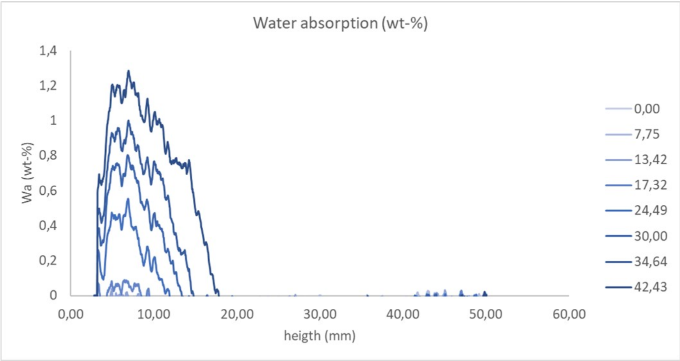

Schlaitdorf Sandstone_C_NC-25C_Sample 2

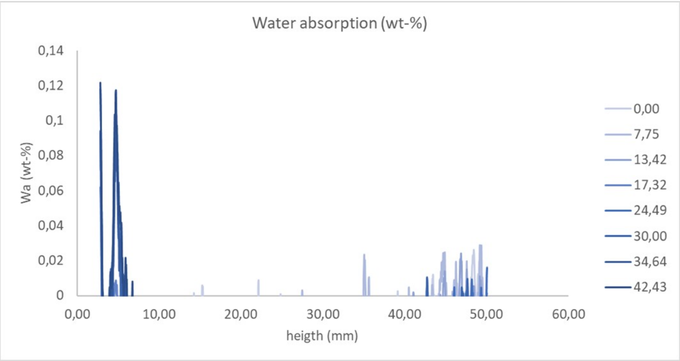

Schlaitdorf Sandstone_C_NC-25C_Sample 3

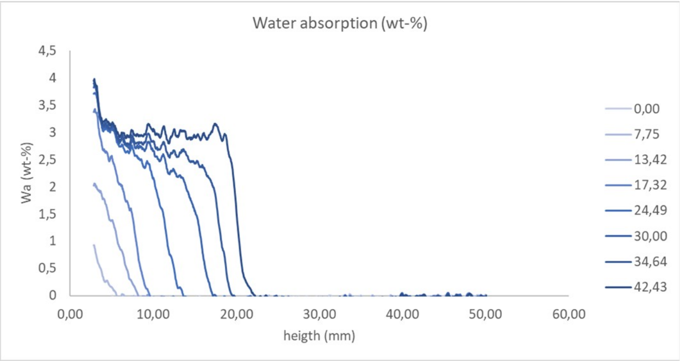

Schlaitdorf Sandstone_C_NC-25C_Oc_Sample 1

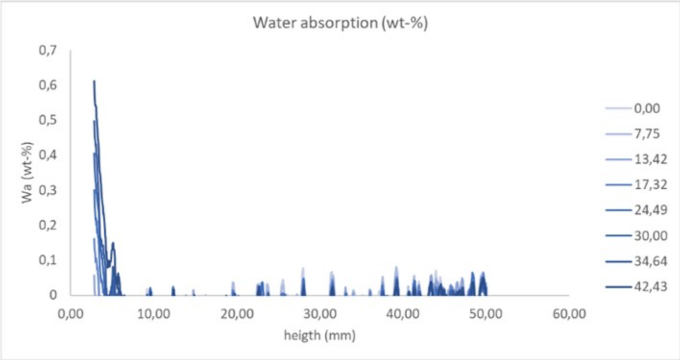

Schlaitdorf Sandstone_C_NC-25C_Oc_Sample 2

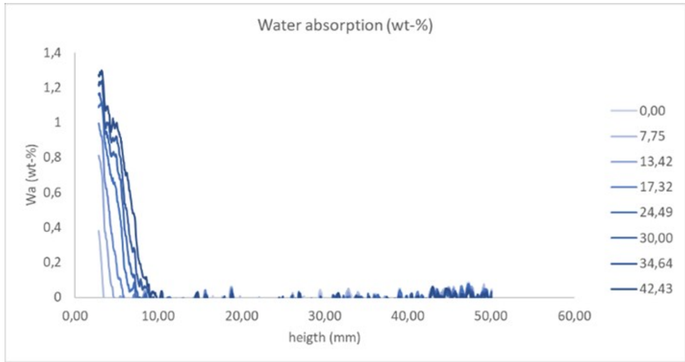

Schlaitdorf Sandstone_C_NC-25C_Oc_Sample 3

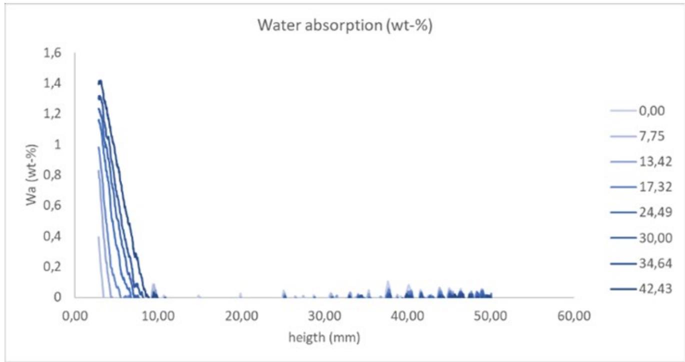

Schlaitdorf Sandstone_B_NC-12C_P_Sample 1

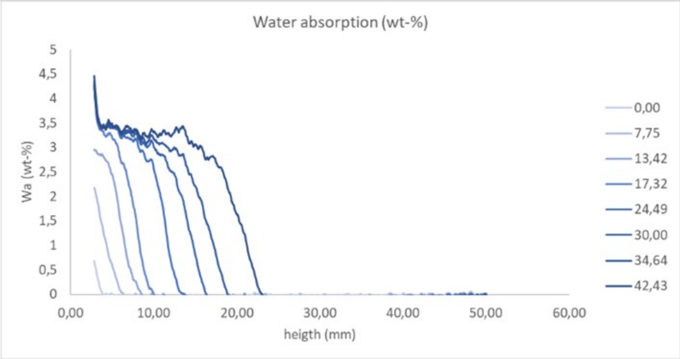

Schlaitdorf Sandstone_B_NC-12C_P_Sample 2

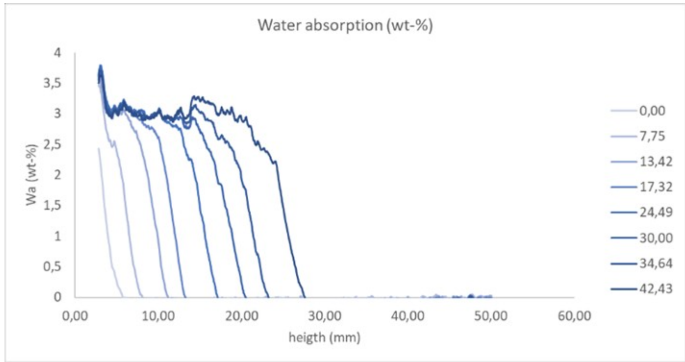

Schlaitdorf Sandstone_B_NC-12C_P_Sample 3

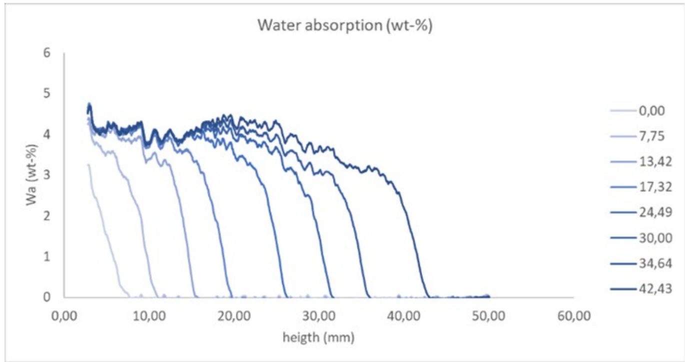

Schlaitdorf Sandstone_B_NC-12C_wP_Sample 1

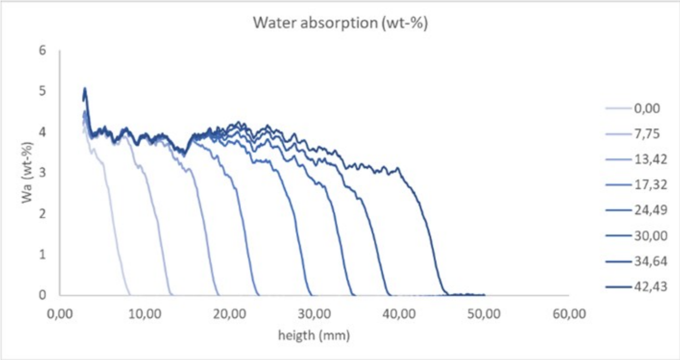

Schlaitdorf Sandstone_B_NC-12C_wP _Sample 2

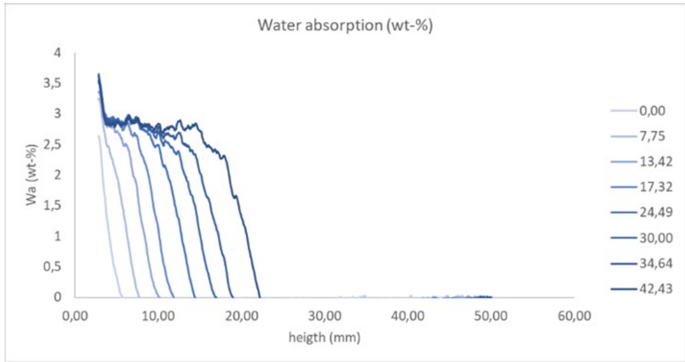

Schlaitdorf Sandstone_B_NC-12C_wP_Sample 3

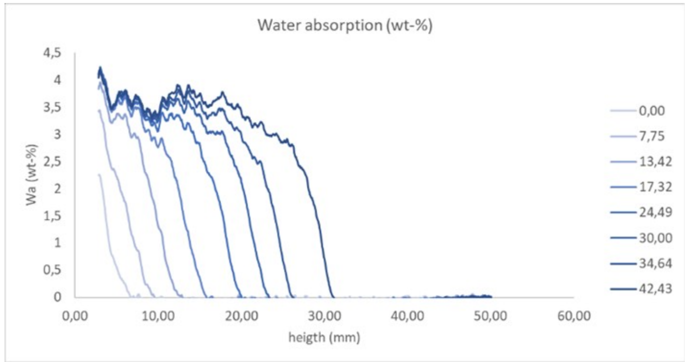

Schlaitdorf Sandstone_B_NC-25C_Sample 1

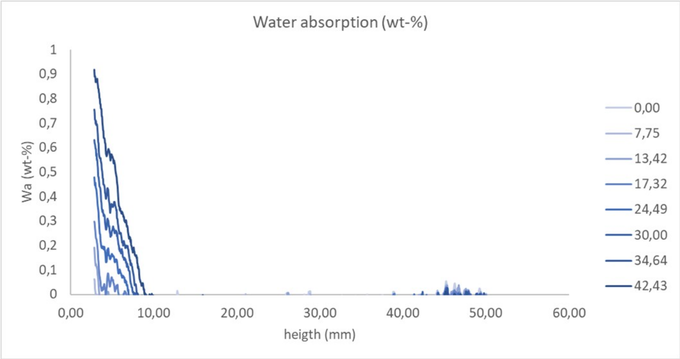

Schlaitdorf Sandstone_B_NC-25C_Sample 2

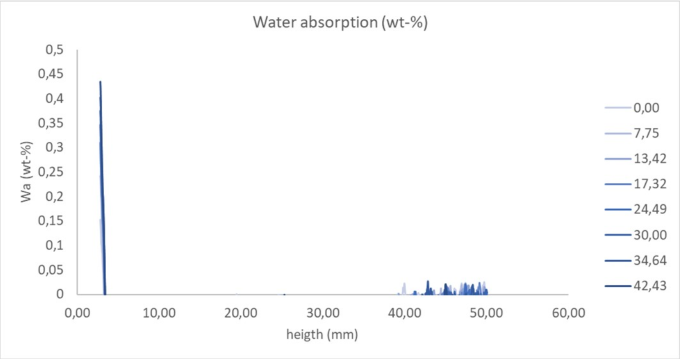

Schlaitdorf Sandstone_B_NC-25C_Sample 3

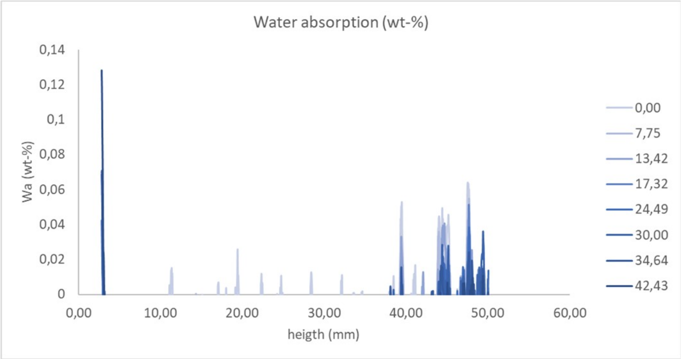

Schlaitdorf Sandstone_P_NC-12C_P_Sample 1

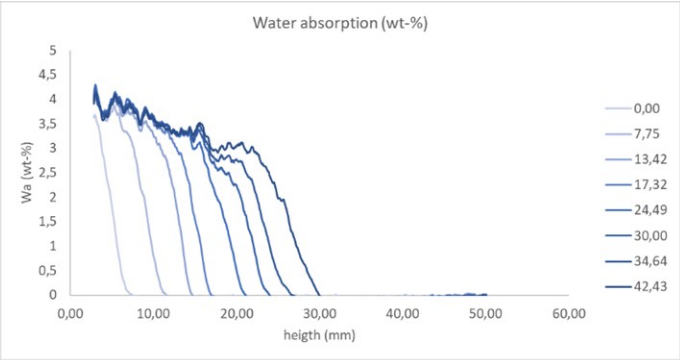

Schlaitdorf Sandstone_P_NC-12C_P _Sample 2

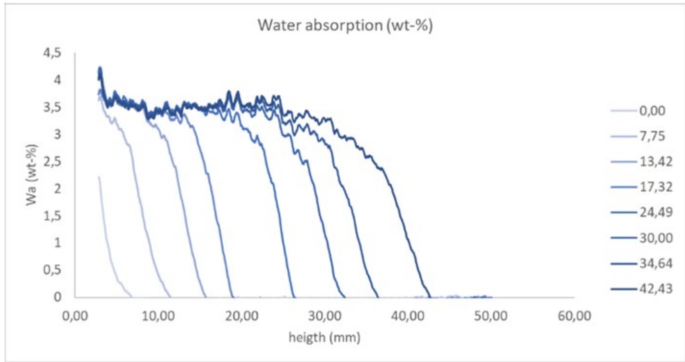

Schlaitdorf Sandstone_P_NC-12C_P_Sample 3

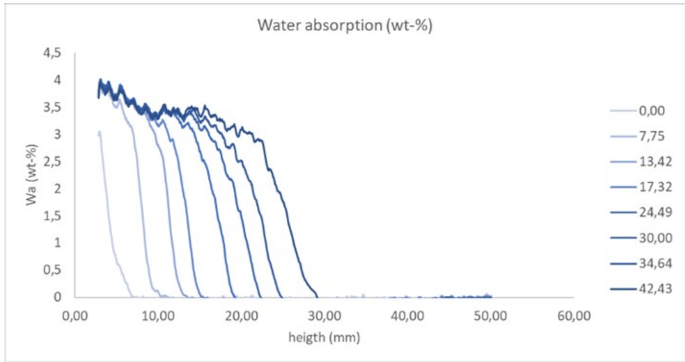

Schlaitdorf Sandstone_P_NC-25C_Sample 1

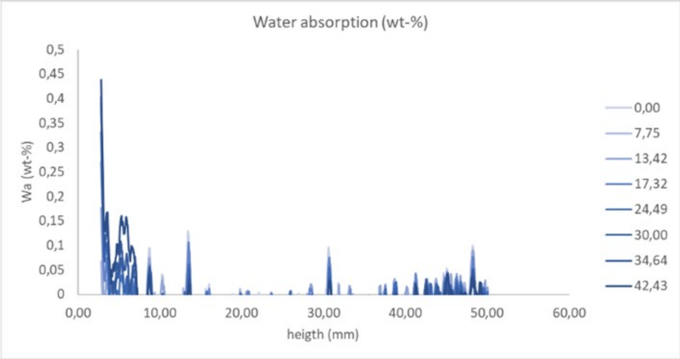

Schlaitdorf Sandstone_P_NC-25C_Sample 2

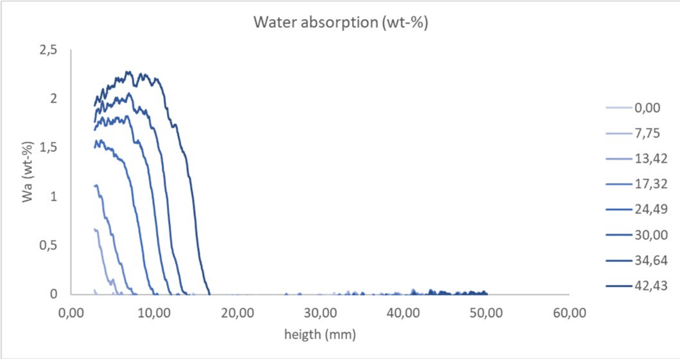

Schlaitdorf Sandstone_P_NC-25C_Sample 3

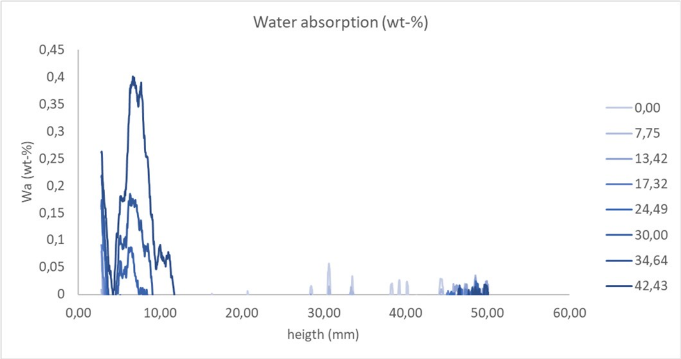

Supplement: Supplementary file 1 [file nanomaterials-09-00635-s001.pdf]
